# Abundance of HPV L1 Intra-Genotype Variants With Capsid Epitopic Modifications Found Within Low- and High-Grade Pap Smears With Potential Implications for Vaccinology

**DOI:** 10.3389/fgene.2019.00489

**Published:** 2019-05-24

**Authors:** Jane Shen-Gunther, Hong Cai, Hao Zhang, Yufeng Wang

**Affiliations:** ^1^Gynecologic Oncology and Clinical Investigation, Department of Clinical Investigation, Brooke Army Medical Center, Fort Sam Houston, TX, United States; ^2^Department of Biology, University of Texas at San Antonio, San Antonio, TX, United States; ^3^South Texas Center for Emerging Infectious Diseases, University of Texas at San Antonio, San Antonio, TX, United States

**Keywords:** human papillomavirus, HPV genotyping, HSIL, late major capsid protein L1, metagenome, next generation sequencing, protein structure prediction, vaccine

## Abstract

**Background:** The aim of this study was to explore the Human Papillomavirus (HPV) genotype composition and intra-genotype variants within individual samples of low- and high-grade cervical cytology by deep sequencing. Clinical, cytological, sequencing, and functional/structural data were forged into an integrated variant profiling pipeline for the detection of potentially vaccine-resistant genotypes or variants.

**Methods:** Low- and high-grade intraepithelial lesion (LSIL and HSIL) cytology samples with +HPV were subjected to amplicon (L1 gene fragment) sequencing by dideoxy (Sanger) and deep methods. Taxonomic, abundance, diversity, and phylogenetic analyses were conducted to determine HPV genotypes/sub-lineages, relative abundance, species diversity and phylogenetic distances within and between samples. Variant detection and functional analysis of translated L1 amino acid sequences determined structural variations of interest.

**Results:** Pure and mixed HPV infections were common among LSIL (*n* = 6) and HSIL (*n* = 6) samples. Taxonomic profiling revealed loss of species richness and gain of dominance by carcinogenic genotypes in HSIL samples. Phylogenetic analysis showed excellent correlation between HPV-type specific genetic distances and carcinogenic potential. For combined LSIL/HSIL samples (*n* = 12), 11 HPV genotypes and 417 mutations were detected: 375 single-nucleotide variants (SNV), 29 insertion/deletion (indel), 12 multi-nucleotide variants (MNV), and 1 replacement variant. The proportion of nonsynonymous mutations was lower for HSIL (0.38) than for LSIL samples (0.51) (*p* < 0.05). HPV variant analysis pinpointed nucleotide-level mutations and amino acid-level structural modifications.

**Conclusion:** HPV L1 intra-host and intra-genotype variants are abundant in LSIL and HSIL samples with potential functional/structural consequences. An integrated multi-omics approach to variant analysis may provide a sensitive and practical means of detecting changes in HPV evolution and dynamics within individuals or populations.

## Introduction

In 1932, Richard Shope isolated the first papillomavirus (PV) from crude extracts of “warty” tumors found on the skin of a wild cottontail rabbit (Shope, 1932). Since then, 183 animal and 225 HPV have been discovered and classified in The Papillomavirus Episteme (PaVE) (Van Doorslaer et al., 2017)^1^ With the advent of metagenomic sequencing, the rate of HPV discovery has accelerated rapidly (Bzhalava et al., 2014) and the resolution of HPV viromes and variants have sharpened immensely (Shen-Gunther et al., 2017) to allow in-depth analysis of genetic variations and functional consequences (van der Weele et al., 2017; Dube Mandishora et al., 2018).

The PV is believed to have co-evolved with their hosts over 350 million years (Doorbar et al., 2015). Through phylogenetic analysis, Chen et al. (2018a) demonstrated that viral niche-adaptation to host ecosystems (tissue tropism) anteceded viral-host codivergence. The PV-host tissue tropism apparently played a vital role in shaping the molecular evolution of oncogenic HPV from archaic hominins to modern humans. HPV-16, an extraordinary result of evolutionary processes over the last 40 million years (Chen et al., 2018a) has emerged as a highly potent carcinogen with a predilection for human mucosa. HPV-16 is now the leading cause of invasive cervical cancer and other cancers of the oropharyngeal and anogenital tracts (Bosch et al., 2013).

The HPV genome is a ∼8,000 base pair (bp), double stranded, circular DNA packaged within a protein capsid. The prototypical genome encodes 6 early genes (E1, E2, E4, E5, E6, and E7) and 2 late genes (L1 and L2) (Van Doorslaer et al., 2017). Specifically, the L1 gene encodes the major capsid protein which forms a pentameric capsomer that self-arranges into a 72-subunit icosahedral capsid. The capsid is essential for viral binding and entry into host-specific tissues (Buck et al., 2013). Furthermore, the L1 coding sequences of the immunogenic surface loops are distinctively poorly conserved due to selective pressures for mutagenesis and immune evasion (Buck et al., 2013).

Recently, whole-genome Sanger and deep sequencing studies have shown a surprisingly high level of intra-host diversity of HPV-16, -18, -52, and -58 (van der Weele et al., 2017, 2018; Hirose et al., 2018). Extensive intra-host HPV L1 sequence variability in 35 HPV genotypes was also discovered in samples from Zimbabwean women by deep sequencing (Dube Mandishora et al., 2018). Such intra-host viral sequence variability is believed to be caused by error-prone host replication machinery used for viral replication and HPV-induced APOBEC deaminase activity with ensuing selective shaping by host tissues and immune responses (Dube Mandishora et al., 2018; Hirose et al., 2018). These remarkable findings of L1 genetic variability are clinically important due to potential structural changes on the epitopes of virions arising from nonsynonymous mutations. The result may be ineffectual binding by host neutralizing antibodies induced by either natural infections or prophylactic vaccines (Bissett et al., 2016; El-Aliani et al., 2017).

Using a multi-omics approach, we aimed to explore the HPV genotype composition and intra-genotype variants within individual samples of low and high-grade cervical cytology. We also focused on the genetic and translated amino acid sequence variations of L1 informed by next-generation sequencing (NGS) for mapping onto the structure of HPV antigenic loops as a means of variant profiling and visualization.

## Materials and Methods

### Subjects and Samples

Residual liquid-based cervical cytology samples were consecutively procured from the Department of Pathology after completion of cytological diagnosis. Demographic and cytohistological data were abstracted from the electronic health record (AHLTA) of the Department of Defense (DoD) and linked to each sample. In our previous study, three categories of samples, i.e., negative for intraepithelial lesion or malignancy (NILM), low-grade squamous intraepithelial lesion (LSIL) and high-grade squamous intraepithelial lesion (HSIL) were collected for HPV genotyping and DNA methylation analysis (Shen-Gunther et al., 2016). For this pilot study, we randomly selected a subset of HPV-positive LSIL (*n* = 6) and HSIL (*n* = 6) for characterization and comparison of viral diversity and variant analysis.

### HPV L1 DNA Amplification and Deep Sequencing

DNA extraction from residual liquid-based cervical cytology for HPV DNA amplification and deep sequencing was performed as described previously (Shen-Gunther et al., 2017). Briefly, HPV DNA was amplified using the consensus primer set: MY09/11 to target a 450 bp region (corresponding to flanking nucleotide positions 6584/7035 on HPV-16) of the L1 gene for genotype identification (Shen-Gunther and Yu, 2011). The PCR products were then purified for construction of DNA libraries using the Nextera XT kit (Illumina). Each DNA sample (1 ng) with a standardized concentration of 0.1–0.2 ng/μL was “tagmented” (fragmented and tagged with sequencing adapters) and barcoded with dual index adaptors. The DNA libraries were normalized quantitatively for equal representation from each sample prior to pooling and sequencing. Paired-end bi-directional sequencing (2 × 300 bp) was performed on the MiSeq (Illumina) instrument using the MiSeq Reagent Kit v3 (600-cycle) for bridge amplification. Quality sequences were subjected to nucleotide BLAST (Altschul et al., 1997) against the HPV sequences in the papillomavirus genome database (PaVE) (Van Doorslaer et al., 2017)^2^, to determine the HPV genotype(s) (Shen-Gunther and Yu, 2011).

The PCR products were concurrently subjected to dideoxy (Sanger) sequencing for validation of deep-sequenced results. Briefly, amplicons (∼200 ng DNA/sample) were sequenced using primer MY11 at Eurofins Operon (United States). The resulting quality sequences were BLAST aligned for HPV genotyping as described above.

### Next-Generation Sequencing (NGS) Data Analysis, Genotyping, and Taxonomic Profiling

The pre-configured, automated Quality Control (QC) workflow implemented in Illumina MiSeq output a series of QC metrics including the summary statistics of the reads, and the Phred quality scores Q which correspond to the base-calling error probabilities (Ewing and Green, 1998; Ewing et al., 1998). The reads were processed using the CLC Genomics Workbench 11.0.1 (QIAGEN). The Core NGS workflow was implemented, including: (1) Preprocessing reads with quality trimming based on quality scores with a limit cutoff 0.05, and the ambiguity number ≤2, and adapter trimming. (2) Merging overlapping pairs to improve the read quality. The parameter setting was mismatch cost 2, gap cost 3, and minimum score 8. (3) Mapping to the nonredundant HPV reference genome database, which was constructed based on the collection and annotation of the PaVE database (Van Doorslaer et al., 2017). Mapping parameters included read alignment match score 1, mismatch penalty 2, linear gap cost for insertion or deletion of 3. (4) Taxonomic profiling. The Microbial Genomics Module was implemented to perform qualification by assigning the read to a HPV genotype if a match is found and quantification of the abundance of each qualified HPV genotype to generate an abundance table for each sample. Reads matching to the host genome were filtered.

### Diversity Analysis of HPV Communities in LSIL and HSIL Samples

The diversity of the HPV genotypes was analyzed for each sample using the Microbial Genomics Module of the CLC Genomics Workbench 11.01.1 (QIAGEN). α-diversity of the HPV communities was computed to measure within-sample variation by (1) the Simpson’s index (Simpson, 1949): $\text{SI}\;=\;1\;-\;\sum_{\text{i}=1}^{n}\;p_{i}^{2}\;$, and (2) Shannon entropy (Shannon, 1948): $\text{H}\;=\;\sum_{1}^{n}\;p_{i}{\text{log}}_{2}p_{\text{i}}$, where *n* was the number of HPV genotypes found in the sample, and *p_i_* was the proportion of reads that were identified as the *i^th^* HPV genotype. β-diversity analysis was performed with the principle coordinate analysis (PCoA) of Bray-Curtis distances (Bray and Curtis, 1957): $B\;=\;\frac{\sum_{\text{i}=1}^{n}\;|x_{i}^{A}\;-\;x_{i}^{B}|}{\sum_{\text{i}=1}^{n}\left(x_{i}^{A}-x_{i}^{B}\right)}$, where *n* is the number of operational taxonomic unit (OTU) *i* and $x_{i}^{A}$ and $x_{i}^{B}$ are the respective abundances of OTU *i* in samples *A* and *B*, to measure the dissimilarity or “distance” of HPV genotype composition between samples. Principal component analysis (PCA) was used to determine the correlative relationship between variables (HPV genotypes) in the LSIL or HSIL group. PCA was performed on the covariance matrix of natural log-transformed abundance data [ln (n +1)] of HPV genotypes within each sample (Rencher and Christensen, 2012). Log transformation was applied to reduce the influence (skewness) of highly abundant genotypes. PCA was performed using STATA/IC 15.0 (StataCorp).

### Phylogenetic Analysis and Tree Construction of HPV Genotypes

Multiple alignment of consensus sequences of each HPV genotype detected in the HSIL and LSIL samples was obtained using the T-coffee program (Notredame et al., 2000). The evolutionary history of the HPV L1 sequences was inferred by using the Maximum Likelihood (ML) method (Felsenstein, 1981) and Tamura-Nei model (Tamura and Nei, 1993). Initial trees for the heuristic search were obtained automatically by applying Neighbor-Joining (NJ) (Saitou and Nei, 1987) and BioNJ (Gascuel, 1997) algorithms to a matrix of pairwise distances estimated using the Maximum Composite Likelihood (MCL) approach, and then selecting the topology with superior log likelihood value. The bootstrap resampling with 1,000 pseudo-replicates was carried out to assess support for each individual branch (Felsenstein, 1985). Bootstrap values of <50% were collapsed and treated as unresolved polytomies. Evolutionary analyses were conducted in MEGA X (Kumar et al., 2018).

### Detection of HPV L1 Sequence Variants, Amino Acid Alterations, and Structural Modifications

Variants were detected by comparing to reference sequences of each HPV type, using the Low Frequency Variant Detection Module in the CLC Bio Genomics Workbench 11.0.1 (QIAGEN), where an error model was included to exclude variants that were likely due to sequencing errors. Variants were classified into four categories: SNV, MNV, indel, or replacement of one or more bases.

The functional consequences of detected variants in each sample were inferred based on the predicted changes at the codon level. These changes were classified as nonsynonymous (with amino acid changes), synonymous (silent mutation without alteration in amino acid designation), or indels which can lead to reading frame shift or early stop codon. To map the amino acid changes to protein structure, BLAST searches were conducted to identify the homologous HPV L1 structure(s) collected in the Protein Data Bank (PDB)^3^ (Berman et al., 2000). 3D models showing the structure of HPV L1 protein with variant and reference sites was created using the CLC Bio Genomics Workbench 11.0.1 (QIAGEN). Another protein structural feature, i.e., surface probability, useful for identification of antigenic determinants was calculated using the protein module of CLC Bio Genomics Workbench 11.0.1 (QIAGEN). The surface probability (accessibility) of an amino acid is predicted using Emini’s formula: *S_n_* = [$\prod_{\text{i}=1}^{6}$ *δ*_n+4-i_]∗(0.37)^-6^ where *S_n_* is the surface probability of amino acid *n* equating to the normalized product of fractional surface probabilities (*δ_x_*) of six amino acids flanked by positions *n* -2 and *n*+ 3 (Emini et al., 1985). The *S_n_* of a random hexapeptide is 1.0 (threshold); a value >1.0 indicates increased surface probability.

### HPV Taxonomy and Carcinogenicity Classifications

The genotype classification of PV is based on the DNA sequence of the L1 gene (de Villiers et al., 2004; Bernard et al., 2010). The definitions for taxonomic ranks (PaVE) are as follows: (1) Genera: members of the same genus share >60% nucleotide sequence identity in the L1 open reading frame (ORF), (2) Species: PV types within a species share between 71 and 89% nucleotide identity within the complete L1 ORF, (3) Genotypes: PV of the same type share ≥90% nucleotide sequence identity, (4) Variants: <2% sequence difference from a known type, (5) Variant lineage: PV genomes with approximately 1.0% nucleotide sequence difference (proposed nomenclature), and (6) Sub-lineage: PV genomes with 0.5–1.0% nucleotide sequence difference (proposed nomenclature).

The World Health Organization (WHO) International Agency for Research on Cancer (IARC) Working Group assessed carcinogenic potential of HPV types and classified them into three categories (International Agency for Research on Cancer, 2012) (1) carcinogenic: HPV types 16, 31, 33, 35, 52, and 58 in α-9, HPV types 18, 39, 45, 59, and 68 in α-7, HPV type 51 in α-5, HPV type 56 in α-6, (2) possibly carcinogenic: HPV types 26, 69, and 82 in α-5, HPV types 30, 53, 66 in α-6, HPV types 70, 85, and 97 in α-7, HPV types 67 in α-9, and HPV types 34 and 73 in α-11, and (3) not classifiable/not carcinogenic: The viruses in this group are from α-1, -2, -3, -4, -8, -10, -13, -14/15. HPV types 6 and 11 were not classifiable, and all others were probably not carcinogenic (Schiffman et al., 2009; Bernard et al., 2010).

## Results

### Deep Sequencing Resolved Viromes and Genotypes of Mixed HPV Infections for Differentiation Between LSIL and HSIL Samples

This study included 12 cytology samples, classified as LSIL (*n* = 6) and HSIL (*n* = 6) (Table 1). The median age of the cohort was 28 years (range, 21–40). For the LSIL group, the median age [34 years (range, 22–40)] was slightly greater than that of the HSIL group [27 years (range, 21–29)]. Histological results from cervical biopsies or excisions were available for 9 of 12 (75%) samples. Histological validation of the cytology samples showed overall good agreement (78%) (Table 1).

**Table 1:** Cytohistological correlation.

	Cytohistological correlation		
**Histology**	**Total**	**LSIL**	**HSIL**

Samples, n	12	6	6
Histology (biopsy or excision)^a^			
Documented, n (%)	9 (75)	4 (67)	5 (83)
Not documented, n (%)	3 (25)	2 (33)	1 (17)
Histological grade^a^			
CIN 0, n (%)	0	0	0
CIN I, n (%)	4 (44)	3 (75)	1 (20)
CIN II/III, n (%)	5 (56)	1 (25)	4 (80)
Cytohistological agreement^b^			
Agreement, %	78		
Expected agreement, %	51		
Kappa	0.55		
Std. Error	0.33		
*p*-value	0.05		

*CIN, cervical intraepithelial neoplasia; HSIL, high-grade squamous intraepithelial lesion; HPV, human papillomavirus; LSIL, low-grade squamous intraepithelial lesion. ^*a*^Cervical histopathology is based on the highest grade documented on cervical biopsy or therapeutic excisional biopsy, i.e., cold knife conization (CKC) and loop excisional procedure (LEEP). Absence or presence of pathology reports in the DoD electronic health records was categorized as “Documented” or “Not documented,” respectively. ^*b*^Cytohistological agreement was calculated using samples with documented histopathology. LSIL cytology corresponds to CIN I histology; HSIL cytology corresponds to CIN II/III histology.*

Both traditional Sanger and NGS platforms were used to detect HPV genotypes and sub-lineages within each sample. Sanger sequencing resolved the single dominant HPV genotype within each sample. Compared to Sanger sequencing, NGS achieved a better resolution in detection of mixed genotypes (up to four in this cohort) and low-abundance genotypes (Table 2). Comparing the dominant genotypes and sub-lineages derived from both sequencing methods, the inter-assay agreement was 100%. Tabulated summary of NGS reads is shown in Table 2. The median of reads that passed quality check for 12 samples was 328,197. The proportion of merged reads that were mapped to reference HPV genotype (s) ranged from 94.9 to 99.8%.

**Table 2:** HPV L1 sequencing results.

Sample Info and PCR^a^			Dideoxy seq^b^		Deep sequencing^c^											

								HPV genotypes and sub-lineages							
ID	PAP	PCR band (n)	HPV type	IARC carc	Total merged reads (n)	Total mapped reads (n)	Total HPV types (n)	HPV #1	HPV#1 mapped reads (n)	HPV #2	HPV#2 mapped reads (n)	HPV #3	HPV#3 mapped reads (n)	HPV #4	HPV#4 mapped reads (n)
179	HSIL	1	**16** D3	CARC	226981	224923	3	**16** D3	218169	**70** B1	4258	**39** A2	2150		
305	HSIL	1	**53** D1	POSC	447187	434307	4	**53** D1	404329	**18** A5	18828	**66** B1	5095	**70** B1	3197
313	HSIL	1	**16** A4	CARC	462487	456343	1	**16** A4	451363						
319	HSIL	1	**16** A4	CARC	521199	493925	2	**16** A4	466086	**70** B1	24150				
386	HSIL	1	**83**	NC	283025	282650	2	**83**	278890	**16** A4	2015				
399	HSIL	1	**16** A4	CARC	240743	239279	1	**16** A4	238954						
81	LSIL	1	**58** C1	CARC	120075	119929	2	**58** C1	91062	**61**	28293				
137	LSIL	1	**66** A1	CARC	459454	458465	1	**66** A1	457259						
138	LSIL	1	**81**	NC	323374	323167	2	**81**	295600	**83**	24988				
140	LSIL	3	**61**	NC	112792	112662	3	**61**	102636	**39** A1	8525	**62**	1075		
141	LSIL	1	**53** A1	POSC	255851	254268	2	**53** A1	247587	**61**	4781				
160	LSIL	1	**16** A4	CARC	429808	428796	2	**16** A4	417946	**18** A5	10551				

*CARC, carcinogenic HPV; HSIL, high-grade squamous intraepithelial lesion; HPV, human papillomavirus; ID, sample identification; IARC Carc, International Agency for Research on Cancer – classification of carcinogenicity; L1, HPV L1 gene amplified by PCR; LSIL, low-grade squamous intraepithelial lesion; PCR, polymerase chain reaction; POSC, possibly carcinogenic; NC, not classifiable/probably not carcinogenic; Sample ID No., sequentially numbered samples grouped as LSIL or HSIL; PAP, Pap smear; Seq, sequencing. ^*a*^Cytologically derived DNA samples amplified by PCR using consensus primers to target the HPV L1 loci. The number of PCR amplicon bands was determined by high-resolution capillary gel electrophoresis. ^*b*^HPV genotype determined by BLAST alignment after amplicon sequencing (dideoxy method). HPV genotype number in bold and sub-lineage in alphanumeric fonts. ^*c*^HPV genotype determined by BLAST alignment after amplicon sequencing (deep method). HPV genotype number in bold and sub-lineage in alphanumeric fonts.*

### HPV Communities Were Dissimilar Between LSIL and HSIL With Loss of Species Richness and Gain of HPV-16 Dominance in HSIL Samples

**Figure 1:**
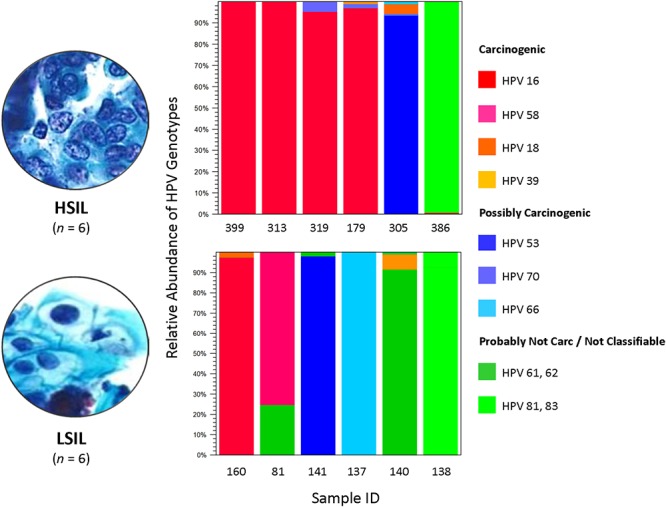
HPV genotype composition found in LSIL and HSIL samples. Deep sequencing of HPV L1 amplicons derived from each LSIL or HSIL sample identified one to four HPV genotypes and quantitated their composition (%) based on number of mapped reads to total mapped reads. Each sample contained a dominant genotype. For HSIL, HPV-16 prevailed in 4 of 6 (67%) samples. In contrast, a mixture of carcinogenic, possibly carcinogenic and probably not carcinogenic HPV genotypes (33% for each category) were identified in LSIL samples. The HPV carcinogenicity is based on IARC’s classification of human carcinogens (International Agency for Research on Cancer, 2012). Carc, carcinogenic; HSIL, high-grade squamous intraepithelial lesion; IARC, International Agency for Research on Cancer; ID, identification; LSIL, low-grade squamous intraepithelial lesion.

The composition of HPV genotypes in each sample is illustrated in Figure 1. For six LSIL L1 samples, the number of genotype(s) per sample was distributed as: 1 (16.7%), 2 (66.6%), and 3 (16.7%). For six HSIL samples, the number of genotype(s) per sample was distributed as: 1 (33.3%), 2 (33.3%), 3 (16.7%), and 4 (16.7%). Notably, all HSIL samples contained at least one carcinogenic HPV genotype, whereas only half of the LSIL samples were found to have a carcinogenic genotype (Table 2).

**Figure 2:**
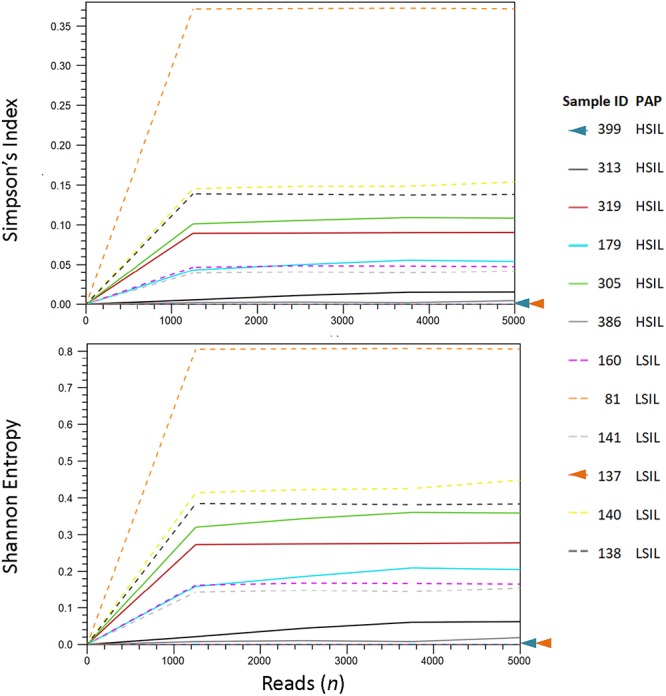
HPV diversity analysis for LSIL and HSIL based on L1 deep sequencing. A total of 10 genotypes out of 6 samples were found in LSIL versus 7 genotypes out of 6 samples for HSIL. The respective Shannon Entropy Indices for LSIL (dashed line) and HSIL samples (solid line) were 0.32 and 0.16, suggesting reduced diversity in HSIL samples. Similarly, species richness measured by Simpson’s index showed a reduction in high-grade cytology (0.12 vs. 0.05 for LSIL and HSIL, respectively). Two samples (137 and 399) contained pure species or zero diversity are indicated by arrowheads. The dominant (most abundant) genotype in LSIL samples was HPV-61 versus HPV-16 for HSIL.

We analyzed the HPV diversity, dominance and community structure between LSIL and HSIL samples. A total of 10 different genotypes were found in single and mixed-infected LSIL samples, whereas seven different genotypes were identified in HSIL samples. The respective Shannon Entropy Indices for LSIL and HSIL samples were 0.32 and 0.16, suggesting reduced diversity in HSIL samples (Figure 2). The dominant (most abundant) genotype in LSIL samples was HPV-61 versus HPV-16 for HSIL. HPV-16, one of the most important carcinogens responsible for almost half of the cervical cancer incidences (Taylor et al., 2016; Mirabello et al., 2017), was found in 5 of 6 (83.3%) HSIL samples. Two additional carcinogenic genotypes HPV-18 and HPV-39 were also discovered in HSIL samples. By contrast, HPV-61, which was considered noncarcinogenic, had 50% occurrence in LSIL samples, indicative of low risk for cervical cancer (Schiffman et al., 2009). It is worthy to note that two LSIL samples contained carcinogenic genotypes (HPV-58 in Sample 81, and HPV-16 in Sample 160), suggesting a finer resolution by HPV molecular profiling than cytological grading for carcinogenic potential.

We further examined the diversity of each sample estimated through read counts and Simpson-Index (Figure 2). The reduced diversity in high grade cytology samples is supported by the mean Simpson’s indices (0.12 versus 0.05 for LSIL and HSIL, respectively). Sample 81 showed a relatively high diversity among LSIL samples, likely due to the presence of two abundant genotypes HPV58 and HPV 61. Sample 305 had the highest diversity in HSIL samples with mixed infection of four genotypes (carcinogenic HPV18, and possibly carcinogenic HPV53, HPV 66, and HPV 70). Samples with pure HPV genotypes, 137 and 399, exhibited low diversity.

**Figure 3:**
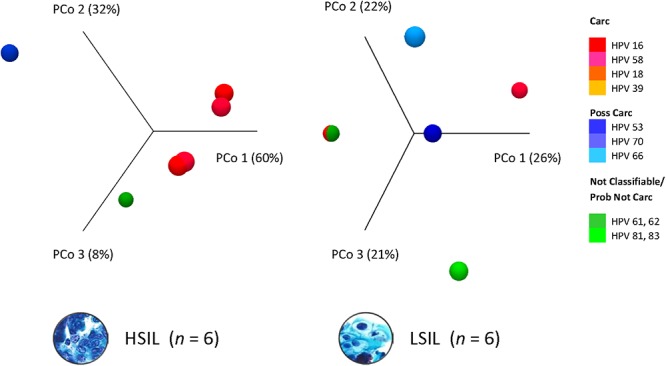
HPV dominance and community structure between LSIL and HSIL. HPV L1 3D-Principal Coordinate Analysis (PCoA) plots of HSIL and LSIL samples showing dissimilarity between the two HPV communities with HPV-16 (PCoA 1) being the most influential genotype in HSIL versus HPV-61 for LSIL (PCoA 1). β-diversity was measured by Bray-Curtis index. Carc, carcinogenic; HSIL, high-grade squamous intraepithelial lesion; ID, identification; LSIL, low-grade squamous intraepithelial lesion.

Dissimilarity of HPV communities across HSIL and LSIL samples was visualized by principle coordinate analysis (PCoA) of Bray-Curtis distances (Bray and Curtis, 1957; Figure 3). PCoA showed HPV-16 (PCo 1, 60%) as being the most influential genotype in HSIL. In contrast, LSIL was influenced about equally (PCo 1–3, 21–26%) by carcinogenic, possibly carcinogenic, and probably not carcinogenic/not classifiable genotypes. As for the PCA results, the component loadings plot for LSIL and HSIL showed the correlative relationship between HPV genotypes along the first two principal components axes (PC1 and PC2) (Supplementary Figure 3). The sum of PC1 and PC2 explained 51.6 and 96.2% of the total variance for LSIL and HSIL, respectively. Comparing LSIL and HSIL, HPV-16 emerged from all other genotypes as the dominant component in HSIL. The score variables plots displayed each sample’s contribution to the principal components. HSIL compared to LSIL had a preponderance of samples containing a high composition of HPV-16.

### Molecular Taxonomy of HPV Genotypes Based on NGS Is Highly Discriminatory and Correlated With IARC-Defined Carcinogenicity

**Figure 4:**
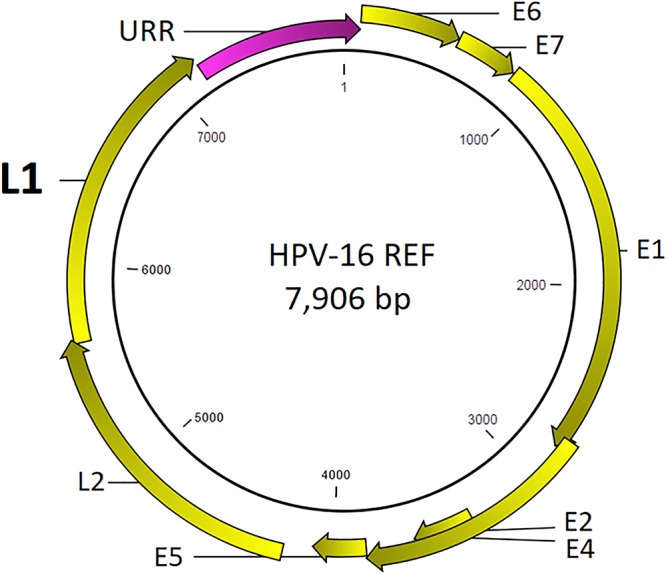
Prototypical HPV genome based on the genetic information of HPV-16 (GenBank ID: K02718). The L1 (450 bp) gene segment of each sample was the target used for sequencing, genotyping, and phylogenetic analysis.

**Figure 5:**
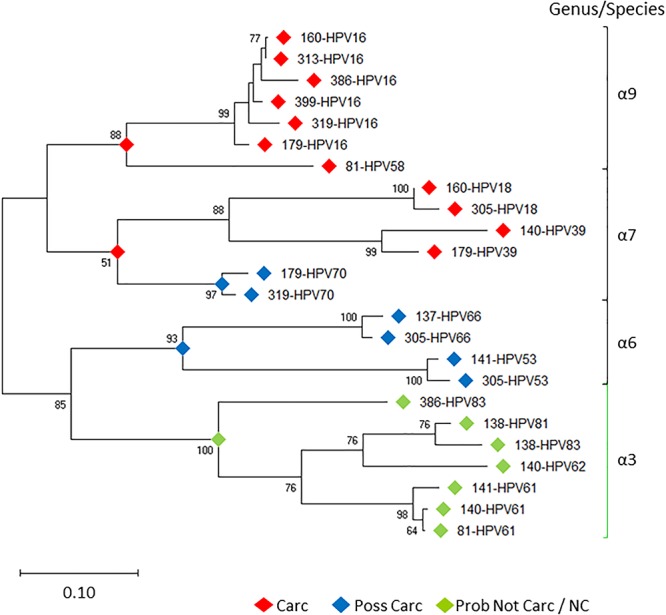
Evolutionary relationships of HPV L1 sequences derived from LSIL and HSIL samples. Phylogenetic tree of L1 nucleotide sequences revealed clades of species (-6, 7, 9) [black bracket] and -3 [green bracket] within the -genus, correlating with the level of IARC-defined carcinogenicity. Between individual samples (sample ID-HPV*n*), HPV intra-type genetic differences are prevalent as distinguished by nonoverlapping branches. The evolutionary history of the HPV L1 sequences was inferred by using the Maximum Likelihood method (Felsenstein, 1981) and Tamura-Nei model (Tamura and Nei, 1993). The tree with the highest log likelihood (-7492.54) is shown. The percentage of trees in which the associated taxa clustered together is shown next to the branches. Initial tree(s) for the heuristic search were obtained automatically by applying Neighbor-Joining (Saitou and Nei, 1987) and BioNJ (Gascuel, 1997) algorithms to a matrix of pairwise distances estimated using the Maximum Composite Likelihood (MCL) approach, and then selecting the topology with superior log likelihood value. Bootstrap resampling with 1,000 pseudo-replicates was carried out to assess support for each individual branch (Felsenstein, 1985). The tree is drawn to scale, with branch lengths measured in the number of substitutions per site. Evolutionary analyses were conducted in MEGA X (Kumar et al., 2018). Carc, carcinogenic; NC, not classifiable; Poss Carc, possibly carcinogenic; Prob Not Carc, probably not carcinogenic; REF, reference genome; URR, upstream regulatory region.

Prototypical HPV genome based on the genetic information of HPV-16 (GenBank ID: K02718) is created using the CLC Bio Genomics Workbench 11.0.1 (QIAGEN) and shown in Figure 4. The L1 (450 bp) gene fragment of each sample was the target used for sequencing, genotyping, and phylogenetic analysis. A maximum likelihood tree was inferred from the L1 sequences derived from single and multi-infected samples (Figure 5). The tree topology is consistent with the HPV species trees (Schiffman et al., 2009; Bernard et al., 2010; International Agency for Research on Cancer, 2012). These L1 sequences were clustered into four clades with strong bootstrap support: (1) α-9 clade included HPV-16 from four HSIL and two LSIL samples, and HPV-58 from an LSIL sample 81. Both HPV-16 and HPV-58 are carcinogenic. (2) α-7 clade included carcinogenic HPV-18 and HPV-39, and a possibly carcinogenic HPV-70, which were shown in three mixed-infected HSIL samples. (3) α-6 clade included possibly carcinogenic HPV-53 and HPV-66. (4) α-3 clade included all the probably not carcinogenic genotypes found in HSIL and LSIL samples (Chen et al., 2018b). Clearly, the broad categorical grade designation based on precancerous cervical lesions (HSIL versus LSIL) was imprecise at predicting carcinogenicity. Conversely, the molecular taxonomy based on NGS is highly discriminatory and correlated well with IARC-defined carcinogenicity.

### Sequence and Structural Variations Identified at HPV Antigenic Sites May Alter Viral Recognition by Innate or Vaccine-Induced Host Defense

**Figure 6:**
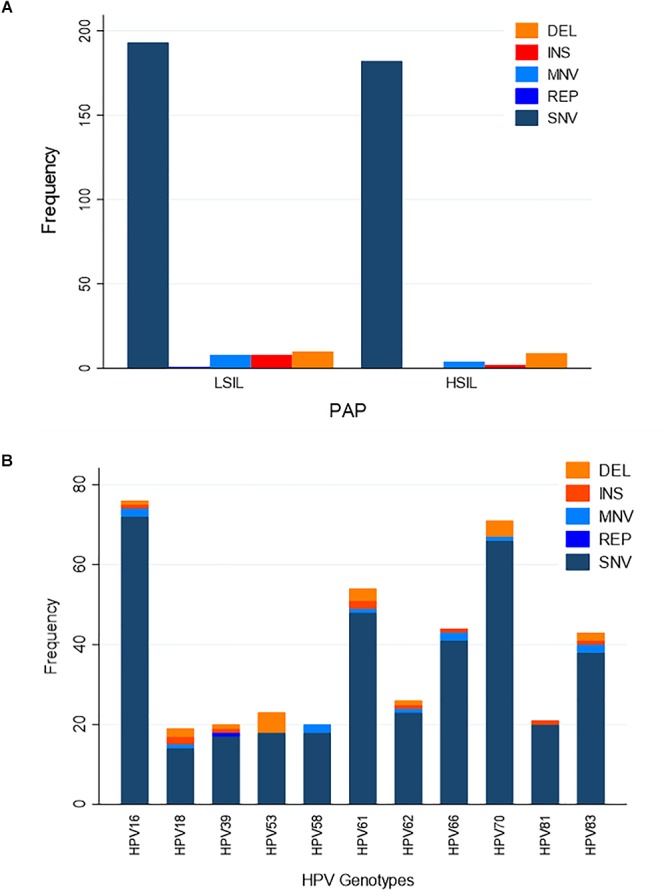
Distribution of variants in HPV L1 sequences. **(A)** Distribution of variants by Pap grade. The predominant type of variants identified in LSIL (*n* = 6) and HSIL (*n* = 6) samples was single nucleotide variant (SNV). **(B)** Distribution of variants by HPV genotype. Eleven HPV genotypes were identified in the deep sequenced LSIL/HSIL (*n* = 12) samples. The top three genotypes with the highest total number of variants were HPV-16, -61, and -70. DEL, deletion; INS, insertion; MNV, multi-nucleotide variant (two or more SNVs in succession); REP, replacement; SNV, single nucleotide variant.

**Figure 7:**
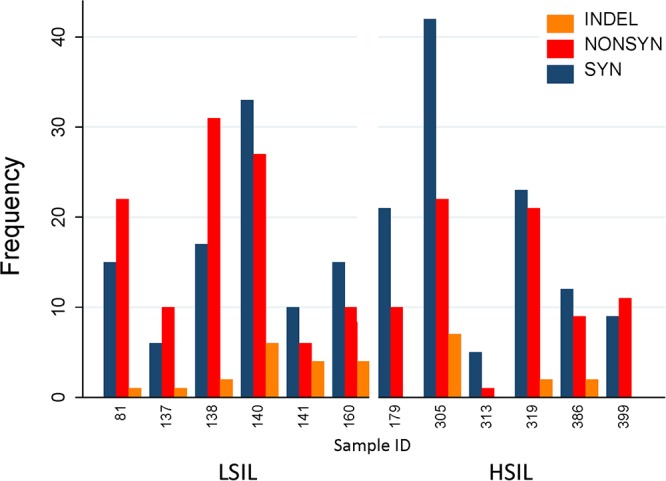
Distribution of variants in HPV L1 sequences found in HSIL and LSIL samples. Variants are categorized as nonsynonymous, synonymous, and insertion/deletion and displayed as frequency counts. The proportion of nonsynonymous mutations was lower for HSIL (0.38) than for LSIL samples (0.51) (*p* = 0.017, Fisher’s exact test). This finding suggests that the inherent competitive advantage of carcinogenic HPV genotypes, e.g., HPV-16 further shaped by intra-host selection may contribute to viral carcinogenesis. One replacement variant of HPV-39 discovered in Sample 140 is not shown in the figure. The annotated variant table including predicted amino acid changes is presented in Supplementary Table 1. HSIL, high-grade squamous intraepithelial lesion; ID, identification; INDEL, insertion/deletion; LSIL, low-grade squamous intraepithelial lesion; NONSYN, nonsynonymous; SYN, synonymous.

We hypothesized that variation in HPV L1 within and among the clinical samples can reveal critical details about the genetic basis for evolution of HPV immune evasion and host-pathogen interactions, because L1 encodes the major capsid protein that plays an important role in virion attachment and entry to the host (Knappe et al., 2007; Dasgupta et al., 2011; Surviladze et al., 2015; Chabeda et al., 2018). Being a natural antigen, the capsid surface is the target of HPV prophylactic vaccines (Harper, 2009; Harper and Williams, 2010; Yang et al., 2016). Supplementary Table 1 lists the position, predicted mutation type and change at the coding region for HPV variants, compared to the respective reference HPV types. For the combined LSIL/HSIL samples (*n* = 12), a total of 417 mutations were detected, including 375 SNVs, 29 indels, 12 MNVs, and one replacement variant. The distribution of these variants for the 12 samples by Pap grade and HPV genotype is shown in Figure 6A,B, respectively. The proportion of nonsynonymous mutations was lower for HSIL (0.38) than for LSIL samples (0.51) (*p* = 0.017, Fisher’s exact test) (Figure 7). On the other hand, probably or probably not carcinogenic HPV types in LSIL samples appeared to be under relaxed functional constraint to accumulate mutations. The distribution of variants in HPV L1 by amino acid positions according to HPV genotype is shown in Supplementary Figure 1.

**Figure 8:**
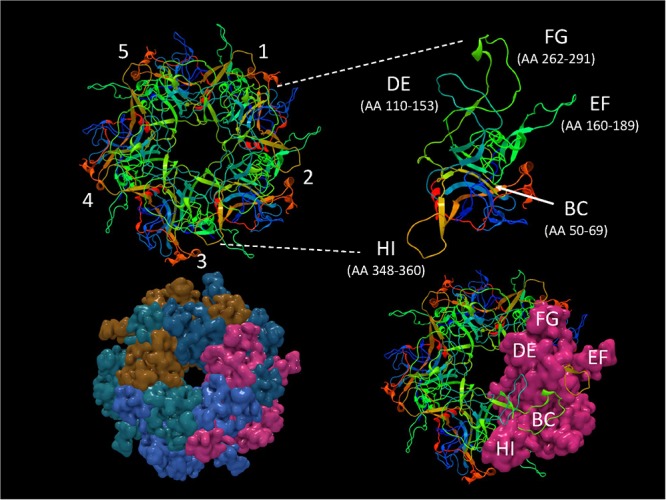
Structure of HPV-16 L1 capsomer. Structural model of HPV-16 L1 capsomer reconstructed from the coordinates and crystal structure filed in Protein Data Bank (PDB ID: 2R5H) (Bishop et al., 2007). The capsomer composed of five L1 subunits (numbered 1–5) are displayed in backbone and surface views to highlight the hypervariable surface loops: BC, DE, EF, FG, and HI and amino acid (AA) positions. These loops are antigenic regions of interest in vaccinology.

**Figure 9:**
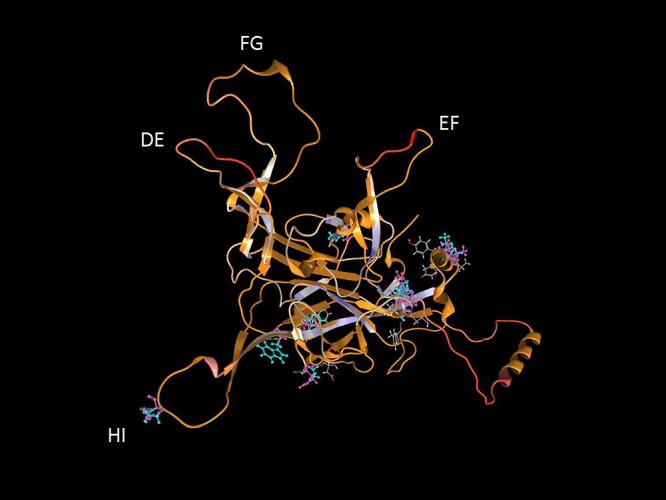
Structural location of L1 variants. Visualization of L1 variants from a HSIL sample (Sample 179) linked to a 3D protein structure. The reference structure is a HPV 16 L1 monomer with accession number 1DZL (Chen et al., 2000) shown in backbone representation. Variant consequences in 3D are identified by the variant in cyan collocated on top of the reference amino acid in purple with attention toward the surface loops. AA, amino acid position.

It is important to identify mutations that is potentially driven by vaccine- or natural infection-induced host immune response. To visualize mutations in 3D, first the structural model of HPV-16 L1 (PDB ID: 2R5H) (Bishop et al., 2007) was reconstructed with demarcated hypervariable surface loops: BC, DE, EF, FG, and HI (Figure 8). Additionally, the HPV-16 L1 protein sequence with surface probability plot for prediction of antigenic determinants on surface proteins (Emini et al., 1985) is provided in Supplementary Figure 2. In the case of HSIL Sample 179, we identified seven nonsynonymous mutations in HPV-16. Figure 9 shows 3D conformational changes visualized by overlying the mutated amino acid residues (cyan) to those (purple) in the reference HPV-16 structure (PDB ID: 1DZL) (Chen et al., 2000). It is particularly noticeable that the mutation at position 353 corresponded to a threonine to proline change (T353P) located at the HI-Loop. The T353P change also increased the surface probability from 3.40 to 3.63 (range, 0–6.47; threshold = 1.0) (Supplementary Figure 2). HI Loop is one of the loops in L1 protein that extends to the outer surface of the capsid complex (Chen et al., 2000; Bishop et al., 2007). This hypervariable HI loop (AA 339–365) contains an HPV-16 immunodominant epitope (Christensen et al., 2001). As seen in human Influenza virus, antigen drift, where mutations are accumulated in antigenic sites, is a potent force driving the evolution of immune evasion and reduced vaccine efficacy (Fitch et al., 1997; Bush et al., 1999; Smith et al., 2004). Similarly, codon changes like T353P at the antigenic regions may confer selective advantage by increasing the likelihood of immune evasion. In addition to T353P, other mutations in this sample may lead to changes in the secondary structure, including W325C at G2 β-sheet, G367P at β-I sheet, T389S at α-2 helix, L441I at a β-turn, Q461P and F462Y near α-5 helix (Bishop et al., 2007).

## Discussion

This study revealed the complex genetic diversity of HPV viromes within low- and high-grade Pap samples. Both pure and mixed infections were common as shown by deep amplicon sequencing. Taxonomic profiling revealed the difference between LSIL and HSIL viral communities with loss of species richness and gain of dominance by carcinogenic genotypes, particularly HPV-16, in HSIL samples. Deep sequencing allowed the detection of carcinogenic HPVs constituting a minor component of a virome which was undisclosed by Sanger sequencing or cytological grading. Phylogenetic inference of the patient-derived L1 sequences showed excellent correlation between HPV type-specific distances and IARC-defined carcinogenic potential. Together with taxonomic profiling, this “Taxo-Phylo” approach holds promise as a molecular taxonomy-based classifier of cervical cytology.

HPV variant detection and analysis pinpointed the nucleotide-level mutations and potential functional, as well as, structural consequences. Localizing mutations to primary sequences and structures can help understand the functional consequence of mutations and identify causal or adaptive mutations. Furthermore, *in silico* modeling of mutations may direct laboratory testing and confirmation of its significance through antigen-antibody binding assays. For example, hepatitis B virus (HBV) genotypes are known to vary by ethno-geography. Mutations in the major hydrophilic regions (MHR) of the hepatitis B surface antigen (HBSAg) have resulted in stable, vaccine-escape mutant virions that are infectious and pathogenic (Carman et al., 1990; Gencay et al., 2018). Recently, investigators have used ultra-deep sequencing and clinical immunoassays (monoclonal antibodies) to detect single-nucleotide, vaccine-escape mutations and associated changes in the HBSAg amino acid residues in clinical samples (Gencay et al., 2018). Similarly, liquid-based cervical cytology samples may be interrogated by deep sequencing and multiplexed immunoassays, e.g., Luminex xMAP^®^ (Peters et al., 2013) to survey HPV L1 mutant virions that may escape from innate or vaccine-induced immunity.

Longitudinal HPV metagenomic surveillance may also provide a sensitive means of detecting changes in HPV evolution and dynamics within individuals or populations. This is clinically important because virulent genotype(s) of low abundance may later dominate the virome if it is inherently more carcinogenic or confers a selective advantage with ensuing clonal expansion. Current published literature on HPV L1 variant analysis is scarce. As noted previously, a high intra-type L1 sequence variability was discovered in 35 HPV genotypes by deep sequencing. These investigators also found unique genotypes and its variants associating with distinct anatomical sites supporting the notion of viral niche-adaptation as shapers of viral evolution (Chen et al., 2018a; Dube Mandishora et al., 2018). However, functional consequences of these mutations were not studied. Another investigation found multiple mutations within the L1 fragment of HPV-16 (MY09/11-primed amplicons) of 35 invasive cervical cancer samples from Morocco (El-Aliani et al., 2017). A distinct mutation in the HI loop (T389P) found in 51.4% of cases could potentially interact with vaccine-induced neutralizing antibodies (El-Aliani et al., 2017). In view of this information, our results are highly consistent with the findings of high intra-host and intra-type L1 sequence variability that could potentially impact vaccine efficacy.

The strength of this study lies in the multi-omics approach developed herein. Integration of clinical metadata, genomic data, and functional/structural information to reveal patient-specific metagenomic profiles and variant structures in 3D is novel and practical. Such individualized virome profiling may provide guidance to clinicians on the risk of cervical cancer and potentially deleterious viral variants/mutations. We acknowledge that our study has limitations in that the sample size was small and a fragment of L1 was studied so overreaching generalizable conclusions cannot be drawn. However, an integrated, holistic approach was established from this dataset to further HPV metagenomics research. Our future direction will be to conduct a large scale, whole-genome or full-sequence L1 variant analysis to survey type-specific variant patterns by cytological grades.

## Conclusion

In this pilot study, NGS provided a cost-effective platform for an unbiased discovery of HPV communities in clinical samples. The HPV genotype composition was shown to be correlated with clinical severity and the carcinogenic risk for cervical cancer. Multi-omics analyses afforded an unprecedented opportunity to better characterize the L1 complexity in clinical samples. Ultimately, this approach will lead to greater understanding of the dynamic interplay between virus and host in HPV pathogenesis.

## Author’s Note

This paper has undergone PAO review at Brooke Army Medical Center and was cleared for publication. The opinions or assertions contained herein are the private views of the authors and are not to be construed as official or reflecting the views of the U.S. Department of the Army, U.S. Department of Defense, or the U.S. government.

## Ethics Statement

This study was approved by the institutional review board of Brooke Army Medical Center, Fort Sam Houston, Texas.

## Author Contributions

JS-G and YW conceived and designed the study and participated in the acquisition of data. JS-G, YW, HC, and HZ analyzed and interpreted the data. JS-G, YW, and HC wrote the manuscript. All authors read and approved the final manuscript.

## Conflict of Interest Statement

The authors declare that the research was conducted in the absence of any commercial or financial relationships that could be construed as a potential conflict of interest.

## Footnotes

1 https://pave.niaid.nih.gov

2 https://pave.niaid.nih.gov

3 https://www.rcsb.org/

## Abbreviations

BLAST: Basic Local Alignment Search Tool
CIN: cervical intraepithelial neoplasia
HPV: Human Papillomavirus
HSIL: high-grade squamous intraepithelial lesion
IARC: International Agency for Research on Cancer
Indel: insertion/deletion
LSIL: low-grade squamous intraepithelial lesion
MCL: Maximum Composite Likelihood
ML: Maximum Likelihood
MNV: multi-nucleotide variant
NGS: next-generation sequencing
NJ: Neighbor-Joining
ORF: open reading frame
Pap: Papanicolaou smear
PaVE: papillomavirus genome database
PCoA: principal coordinate analysis
PDB: Protein Data Bank
QC: Quality Control (QC)
SNV: single nucleotide variant
WHO: World Health Organization.

## References

[B1] Altschul S. F. Madden T. L. Schaffer A. A. Zhang J. Zhang Z. Miller W. (1997). Gapped BLAST and PSI-BLAST: a new generation of protein database search programs. *Nucleic Acids Res.* 25 3389–3402. 10.1093/nar/25.17.3389 9254694 PMC146917

[B2] Berman H. M. Westbrook J. Feng J. Gilliland G. Bhat T. N. Weissig H. (2000). The protein data bank. *Nucleic Acids Res.* 28 235–242. 10592235 10.1093/nar/28.1.235 PMC102472

[B3] Bernard H. U. Burk R. D. Chen Z. van Doorslaer K. zur Hausen H. de Villiers E. M. (2010). Classification of papillomaviruses (PVs) based on 189 PV types and proposal of taxonomic amendments. *Virology* 401 70–79. 10.1016/j.virol.2010.02.002 20206957 PMC3400342

[B4] Bishop B. Dasgupta J. Klein M. Garcea R. L. Christensen N. D. Zhao R. (2007). Crystal structures of four types of human papillomavirus L1 capsid proteins: understanding the specificity of neutralizing monoclonal antibodies. *J. Biol. Chem.* 282 31803–31811. 10.1074/jbc.m706380200 17804402

[B5] Bissett S. L. Godi A. Beddows S. (2016). The DE and FG loops of the HPV major capsid protein contribute to the epitopes of vaccine-induced cross-neutralising antibodies. *Sci. Rep.* 22:39730. 10.1038/srep39730 28004837 PMC5177933

[B6] Bosch F. X. Broker T. R. Forman D. Moscicki A. B. Gillison M. L. Doorbar J. (2013). Comprehensive control of human papillomavirus infections and related diseases. *Vaccine* 31(Suppl. 5), F1–F31. 10.1016/j.vaccine.2013.10.001 24331745

[B7] Bray J. R. Curtis J. T. (1957). An ordination of the upland forest communities of Southern Wisconsin. *Ecol. Monogr.* 27 326–349.

[B8] Buck C. B. Day P. M. Trus B. L. (2013). The papillomavirus major capsid protein L1. *Virology* 445 169–174. 10.1016/j.virol.2013.05.038 23800545 PMC3783536

[B9] Bush R. M. Fitch W. M. Bender C. A. Cox N. J. (1999). Positive selection on the H3 hemagglutinin gene of human influenza virus A. *Mol. Biol. Evol.* 16 1457–1465. 10.1093/oxfordjournals.molbev.a026057 10555276

[B10] Bzhalava D. Mühr L. S. Lagheden C. Ekström J. Forslund O. Dillner J. (2014). Deep sequencing extends the diversity of human papillomaviruses in human skin. *Sci. Rep.* 24:5807. 10.1038/srep05807 25055967 PMC4108911

[B11] Carman W. F. Zanetti A. R. Karayiannis P. Waters J. Manzillo G. Tanzi E. (1990). Vaccine-induced escape mutant of hepatitis B virus. *Lancet* 11 325–329. 10.1016/0140-6736(90)91874-a 1697396

[B12] Chabeda A. Yanez R. J. R. Lamprecht R. Meyers A. E. Rybicki E. P. Hitzeroth I. I. (2018). Therapeutic vaccines for high-risk HPV-associated diseases. *Papillomavirus Res.* 5 46–58. 10.1016/j.pvr.2017.12.006 29277575 PMC5887015

[B13] Chen X. S. Garcea R. L. Goldberg I. Casini G. Harrison S. C. (2000). Structure of small virus-like particles assembled from the L1 protein of human papillomavirus 16. *Mol. Cell.* 5 557–567. 10.1016/s1097-2765(00)80449-9 10882140

[B14] Chen Z. DeSalle R. Schiffman M. Herrero R. Wood C. E. Ruiz J. C. (2018a). Niche adaptation and viral transmission of human papillomaviruses from archaic hominins to modern humans. *PLoS Pathog.* 14:e1007352. 10.1371/journal.ppat.1007352 30383862 PMC6211759

[B15] Chen Z. Schiffman M. Herrero R. DeSalle R. Anastos K. Segondy M. (2018b). Classification and evolution of human papillomavirus genome variants: alpha-5 (HPV26, 51, 69, 82), Alpha-6 (HPV30, 53, 56, 66), Alpha-11 (HPV34, 73), Alpha-13 (HPV54) and Alpha-3 (HPV61). *Virology* 516 86–101. 10.1016/j.virol.2018.01.002 29331867 PMC6093212

[B16] Christensen N. D. Cladel N. M. Reed C. A. Budgeon L. R. Embers M. E. Skulsky D. M. (2001). Hybrid papillomavirus L1 molecules assemble into virus-like particles that reconstitute conformational epitopes and induce neutralizing antibodies to distinct HPV types. *Virology* 291 324–334. 10.1006/viro.2001.1220 11878901

[B17] Dasgupta J. Bienkowska-Haba M. Ortega M. E. Patel H. D. Bodevin S. Spillmann D. (2011). Structural basis of oligosaccharide receptor recognition by human papillomavirus. *J. Biol. Chem.* 286 2617–2624. 10.1074/jbc.M110.160184 21115492 PMC3024757

[B18] de Villiers E.-M. Fauquet C. Broker T. R. Bernard H.-U. zur Hausen H. (2004). Classification of papillomaviruses. *Virology* 324 17–27. 10.1016/j.virol.2004.03.033 15183049

[B19] Doorbar J. Egawa N. Griffin H. Kranjec C. Murakami I. (2015). Human papillomavirus molecular biology and disease association. *Rev. Med. Virol.* 25(Suppl.1), 2–23. 10.1002/rmv.1822 25752814 PMC5024016

[B20] Dube Mandishora R. S. Gjøtterud K. S. Lagström S. Stray-Pedersen B. Duri K. Chin’ombe N. (2018). Intra-host sequence variability in human papillomavirus. *Papillomavirus Res.* 5 180–191. 10.1016/j.pvr.2018.04.006 29723682 PMC6047465

[B21] El-Aliani A. Alaoui M. A. E. Chaoui I. Ennaji M. M. Attaleb M. Mzibri M. E. (2017). Naturally occurring capsid protein variants L1 of human papillomavirus genotype 16 in Morocco. *Bioinformation* 13 241–248. 10.6026/97320630013241 28959092 PMC5609288

[B22] Emini E. A. Hughes J. V. Perlow D. S. Boger J. (1985). Induction of hepatitis a virus-neutralizing antibody by a virus-specific synthetic peptide. *J. Virol.* 55 836–839. 2991600 10.1128/jvi.55.3.836-839.1985 PMC255070

[B23] Ewing B. Green P. (1998). Base-calling of automated sequencer traces using phred. II. Error probabilities. *Genome Res.* 8 186–194. 10.1101/gr.8.3.186 9521922

[B24] Ewing B. Hillier L. Wendl M. C. Green P. (1998). Base-calling of automated sequencer traces using phred. I. Accuracy assessment. *Genome Res.* 8 175–185. 10.1101/gr.8.3.175 9521921

[B25] Felsenstein J. (1981). Evolutionary trees from DNA sequences: a maximum likelihood approach. *J. Mol. Evol.* 17 368–376. 10.1007/bf01734359 7288891

[B26] Felsenstein J. (1985). Confidence-limits on phylogenies - an approach using the bootstrap. *Evolution* 39 783–791. 10.1111/j.1558-5646.1985.tb00420.x 28561359

[B27] Fitch W. M. Bush R. M. Bender C. A. Cox N. J. (1997). Long term trends in the evolution of H(3) HA1 human influenza type A. *Proc. Natl. Acad. Sci. U.S.A.* 94 7712–7718. 10.1073/pnas.94.15.7712 9223253 PMC33681

[B28] Gascuel O. (1997). BIONJ: an improved version of the NJ algorithm based on a simple model of sequence data. *Mol. Biol. Evol.* 14 685–695. 10.1093/oxfordjournals.molbev.a025808 9254330

[B29] Gencay M. Vermeulen M. Neofytos D. Westergaard G. Pabinger S. Kriegner A. (2018). Substantial variation in the hepatitis B surface antigen (HBsAg) in hepatitis B virus (HBV)-positive patients from South Africa: reliable detection of HBV by the Elecsys HBsAg II assay. *J. Clin. Virol.* 101 38–43. 10.1016/j.jcv.2018.01.011 29414186

[B30] Harper D. M. (2009). Currently approved prophylactic HPV vaccines. *Expert Rev. Vaccines* 8 1663–1679. 10.1586/erv.09.123 19943762

[B31] Harper D. M. Williams K. B. (2010). Prophylactic HPV vaccines: current knowledge of impact on gynecologic premalignancies. *Discov. Med.* 10 7–17. 20670593

[B32] Hirose Y. Onuki M. Tenjimbayashi Y. Mori S. Ishii Y. Takeuchi T. (2018). Within-host variations of human papillomavirus reveal APOBEC signature mutagenesis in the viral genome. *J. Virol.* 92 e00017-18. 10.1128/JVI.00017-18 29593040 PMC5974501

[B33] International Agency for Research on Cancer (2012). *IARC Monographs on the Evaluation of Carcinogenic Risks to Humans-Human Papillomaviruses.* Geneva: World Health Organization, 255–313.

[B34] Knappe M. Bodevin S. Selinka H. C. Spillmann D. Streeck R. E. Chen X. S. (2007). Surface-exposed amino acid residues of HPV16 L1 protein mediating interaction with cell surface heparan sulfate. *J. Biol. Chem.* 282 27913–27922. 10.1074/jbc.m705127200 17640876

[B35] Kumar S. Stecher G. Li M. Knyaz C. Tamura K. (2018). MEGA X: molecular evolutionary genetics analysis across computing platforms. *Mol. Biol. Evol.* 35 1547–1549. 10.1093/molbev/msy096 29722887 PMC5967553

[B36] Mirabello L. Yeager M. Yu K. Clifford G. M. Xiao Y. Zhu B. (2017). HPV16 E7 genetic conservation is critical to carcinogenesis. *Cell* 170 1164–1174.e6. 10.1016/j.cell.2017.08.001 28886384 PMC5674785

[B37] Notredame C. Higgins D. G. Heringa J. (2000). T-Coffee: a novel method for fast and accurate multiple sequence alignment. *J. Mol. Biol.* 302 205–217. 10.1006/jmbi.2000.4042 10964570

[B38] Peters J. Thomas D. Boers E. de Rijk T. Berthiller F. Haasnoot W. (2013). Colour-encoded paramagnetic microbead-based direct inhibition triplex flow cytometric immunoassay for ochratoxin A, fumonisins and zearalenone in cereals and cereal-based feed. *Anal. Bioanal. Chem.* 405 7783–7794. 10.1007/s00216-013-7095-7 23760139 PMC3765849

[B39] Rencher A. C. Christensen W. F. (2012). *Methods of Multivariate Analysis*, 3rd Edn New Jersey, NJ: John Wiley & Sons, 405–433.

[B40] Saitou N. Nei M. (1987). The neighbor-joining method: a new method for reconstructing phylogenetic trees. *Mol. Biol. Evol.* 4 406–425. 3447015 10.1093/oxfordjournals.molbev.a040454

[B41] Schiffman M. Clifford G. Buonaguro F. M. (2009). Classification of weakly carcinogenic human papillomavirus types: addressing the limits of epidemiology at the borderline. *Infect. Agent Cancer* 4 8. 10.1186/1750-9378-4-8 19486508 PMC2694995

[B42] Shannon C. E. (1948). A mathematical theory of communication. *Bell Syst. Techn. J.* 27 623–656.

[B43] Shen-Gunther J. Wang C. M. Poage G. M. Lin C. L. Perez L. Banks N. A. (2016). Molecular Pap smear: HPV genotype and DNA methylation of ADCY8, CDH8, and ZNF582 as an integrated biomarker for high-grade cervical cytology. *Clin. Epigenet.* 13:96. 10.1186/s13148-016-0263-9 27651839 PMC5022163

[B44] Shen-Gunther J. Wang Y. Lai Z. Poage G. M. Perez L. Huang T. H. (2017). Deep sequencing of HPV E6/E7 genes reveals loss of genotypic diversity and gain of clonal dominance in high-grade intraepithelial lesions of the cervix. *BMC Genomics* 18:231. 10.1186/s12864-017-3612-y 28288568 PMC5348809

[B45] Shen-Gunther J. Yu X. (2011). HPV molecular assays: defining analytical and clinical performance characteristics for cervical cytology specimens. *Gynecol. Oncol.* 123 263–271. 10.1016/j.ygyno.2011.07.017 21839499

[B46] Shope R. E. (1932). A transmissible tumor-like condition in rabbits. *J. Exp. Med.* 30 793–802. 10.1084/jem.56.6.793 19870103 PMC2132208

[B47] Simpson E. H. (1949). Measurement of diversity. *Nature* 163 688–688.

[B48] Smith D. J. Lapedes A. S. de Jong J. C. Bestebroer T. M. Rimmelzwaan G. F. Osterhaus A. D. (2004). Mapping the antigenic and genetic evolution of influenza virus. *Science* 305 371–376. 10.1126/science.1097211 15218094

[B49] Surviladze Z. Sterkand R. T. Ozbun M. A. (2015). Interaction of human papillomavirus type 16 particles with heparan sulfate and syndecan-1 molecules in the keratinocyte extracellular matrix plays an active role in infection. *J. Gen. Virol.* 96 2232–2241. 10.1099/vir.0.000147 26289843 PMC4681067

[B50] Tamura K. Nei M. (1993). Estimation of the number of nucleotide substitutions in the control region of mitochondrial DNA in humans and chimpanzees. *Mol. Biol. Evol.* 10 512–526. 8336541 10.1093/oxfordjournals.molbev.a040023

[B51] Taylor S. Bunge E. Bakker M. Castellsague X. (2016). The incidence, clearance and persistence of non-cervical human papillomavirus infections: a systematic review of the literature. *BMC Infect. Dis.* 16:293. 10.1186/s12879-016-1633-9 27301867 PMC4908763

[B52] van der Weele P. Meijer C. J. L. M. King A. J. (2017). Whole-genome sequencing and variant analysis of human papillomavirus 16 infections. *J. Virol.* 91 e00844-17. 10.1128/JVI.00844-17 28701400 PMC5599754

[B53] van der Weele P. Meijer C. J. L. M. King A. J. (2018). High whole-genome sequence diversity of human papillomavirus type 18 isolates. *Viruses* 10:E68. 10.3390/v10020068 29414918 PMC5850375

[B54] Van Doorslaer K. Li Z. Xirasagar S. Maes P. Kaminsky D. Liou D. (2017). The papillomavirus episteme: a major update to the papillomavirus sequence database. *Nucleic Acids Res.* 45 D499–D506. 10.1093/nar/gkw879 28053164 PMC5210616

[B55] Yang A. Farmer E. Wu T. C. Hung C. F. (2016). Perspectives for therapeutic HPV vaccine development. *J. Biomed. Sci.* 23:75. 10.1186/s12929-016-0293-9 PMC5096309 27809842

