# Supplementary material for: Abundance of HPV L1 Intra-Genotype Variants With Capsid Epitopic Modifications Found Within Low- and High-Grade Pap Smears With Potential Implications for Vaccinology
Source: Front Genet. 2019 May 24;10:489. doi: 10.3389/fgene.2019.00489 (PMC6558378; doi:10.3389/fgene.2019.00489)
Supplement: Supplementary file 1 [file Data_Sheet_1.PDF]

Supplementary Table 1. HPV L1 variants detected in LSIL and HSIL Pap samples[a]

Sample ID	Pap grade	IARC Carc	Mapping	Ref Position	Type	Ref A	Allele	Count	Coverage	Freq	Avg Quality	Coding change	Amino acid change
179	HSIL	CARC	HPV16REF mapping	6592	SNV	C	A	4108	9952	41.28	35.14	HPV16-L1:c.954C>A	
179	HSIL	CARC	HPV16REF mapping	6592	SNV	C	T	5759	9952	57.87	35.19	HPV16-L1:c.954C>T	
179	HSIL	CARC	HPV16REF mapping	6595	SNV	C	T	15067	15096	99.81	36.71	HPV16-L1:c.957C>T	
179	HSIL	CARC	HPV16REF mapping	6598	SNV	T	C	4319	25607	16.87	36.29	HPV16-L1:c.960T>C	
179	HSIL	CARC	HPV16REF mapping	6613	SNV	G	T	518	32896	1.57	37.15	HPV16-L1:c.975G>T	HPV16-L1:p.Trp325Cys
179	HSIL	CARC	HPV16REF mapping	6695	SNV	A	C	49264	49330	99.87	36.83	HPV16-L1:c.1057A>C	HPV16-L1:p.Thr353Pro
179	HSIL	CARC	HPV16REF mapping	6721	SNV	G	A	54444	54693	99.54	36.93	HPV16-L1:c.1083G>A	
179	HSIL	CARC	HPV16REF mapping	6737	SNV	G	T	722	58023	1.24	36.82	HPV16-L1:c.1099G>T	HPV16-L1:p.Gly367Trp
179	HSIL	CARC	HPV16REF mapping	6803	SNV	A	T	56660	56880	99.61	37.21	HPV16-L1:c.1165A>T	HPV16-L1:p.Thr389Ser
179	HSIL	CARC	HPV16REF mapping	6854	SNV	C	T	53307	53432	99.77	36.84	HPV16-L1:c.1216C>T	
179	HSIL	CARC	HPV16REF mapping	6865	SNV	C	T	55702	55957	99.54	36.71	HPV16-L1:c.1227C>T	
179	HSIL	CARC	HPV16REF mapping	6959	SNV	C	A	388	29851	1.30	37.23	HPV16-L1:c.1321C>A	HPV16-L1:p.Leu441Ile
179	HSIL	CARC	HPV16REF mapping	6970	SNV	C	T	28711	28777	99.77	37.17	HPV16-L1:c.1332C>T	
179	HSIL	CARC	HPV16REF mapping	6994	SNV	G	A	22678	22808	99.43	37.22	HPV16-L1:c.1356G>A	
179	HSIL	CARC	HPV16REF mapping	7020	SNV	A	C	162	13522	1.20	36.97	HPV16-L1:c.1382A>C	HPV16-L1:p.Gln461Pro
179	HSIL	CARC	HPV16REF mapping	7023	SNV	T	A	4869	12188	39.95	35.97	HPV16-L1:c.1385T>A	HPV16-L1:p.Phe462Tyr
179	HSIL	CARC	HPV16REF mapping	7027	SNV	T	C	159	833	19.09	35.52	HPV16-L1:c.1389T>C	
179	HSIL	POSC	HPV70REF mapping	6537	SNV	A	T	116	249	46.59	35.29	HPV70-L1:c.948A>T	
179	HSIL	POSC	HPV70REF mapping	6540	SNV	C	T	320	323	99.07	36.76	HPV70-L1:c.951C>T	
179	HSIL	POSC	HPV70REF mapping	6543	SNV	T	C	379	467	81.16	36.80	HPV70-L1:c.954T>C	
179	HSIL	POSC	HPV70REF mapping	6560	SNV	A	G	601	602	99.83	37.09	HPV70-L1:c.971A>G	HPV70-L1:p.His324Arg
179	HSIL	POSC	HPV70REF mapping	6633	SNV	G	A	1058	1058	100.00	37.12	HPV70-L1:c.1044G>A	
179	HSIL	POSC	HPV70REF mapping	6741	SNV	T	A	986	986	100.00	37.26	HPV70-L1:c.1152T>A	
179	HSIL	POSC	HPV70REF mapping	6801	SNV	A	C	1045	1049	99.62	36.75	HPV70-L1:c.1212A>C	
179	HSIL	POSC	HPV70REF mapping	6873	SNV	A	G	903	903	100.00	36.92	HPV70-L1:c.1284A>G	
179	HSIL	POSC	HPV70REF mapping	6886	SNV	A	G	846	847	99.88	36.78	HPV70-L1:c.1297A>G	HPV70-L1:p.Thr433Ala
179	HSIL	POSC	HPV70REF mapping	6971	SNV	T	A	141	205	68.78	36.87	HPV70-L1:c.1382T>A	HPV70-L1:p.Phe461Tyr
179	HSIL	CARC	HPV39REF mapping	6596	SNV	C	T	114	116	98.28	36.65	HPV39-L1:c.954C>T	
179	HSIL	CARC	HPV39REF mapping	6638	SNV	T	A	501	502	99.80	37.18	HPV39-L1:c.996T>A	
179	HSIL	CARC	HPV39REF mapping	6903	SNV	C	T	425	427	99.53	36.92	HPV39-L1:c.1261C>T	
179	HSIL	CARC	HPV39REF mapping	7025	SNV	A	G	110	113	97.35	36.80	HPV39-L1:c.1383A>G	
305	HSIL	CARC	HPV18REF mapping	6566	SNV	T	A	159	1054	15.09	35.86	HPV18-L1:c.954T>A	
305	HSIL	CARC	HPV18REF mapping	6572	SNV	C	T	218	2587	8.43	36.47	HPV18-L1:c.960C>T	
305	HSIL	CARC	HPV18REF mapping	6834	Insertion	-	C	77	3727	2.07	36.32	HPV18-L1:c.1229dup	HPV18-L1:p.Pro411fs
305	HSIL	CARC	HPV18REF mapping	6834	Deletion	C	-	100	3737	2.68	36.54	HPV18-L1:c.1229delC	HPV18-L1:p.Pro410fs
305	HSIL	CARC	HPV18REF mapping	6998	SNV	A	G	1204	1205	99.92	37.03	HPV18-L1:c.1386A>G	
305	HSIL	CARC	HPV18REF mapping	7000	SNV	A	T	318	1205	26.39	36.03	HPV18-L1:c.1388A>T	HPV18-L1:p.Tyr463Phe
305	HSIL	CARC	HPV18REF mapping	7004	SNV	C	T	52	880	5.91	35.13	HPV18-L1:c.1392C>T	
305	HSIL	POSC	HPV53REF mapping	6601	SNV	A	T	407	460	88.48	37.42	HPV53-L1:c.944A>T	HPV53-L1:p.Gln315Leu
305	HSIL	POSC	HPV53REF mapping	6605	SNV	A	T	4143	14721	28.14	35.20	HPV53-L1:c.948A>T	
305	HSIL	POSC	HPV53REF mapping	6605	Deletion	A	-	384	14721	2.61	36.51	HPV53-L1:c.948delA	HPV53-L1:p.His317fs
305	HSIL	POSC	HPV53REF mapping	6611	SNV	T	C	8028	49014	16.38	36.28	HPV53-L1:c.954T>C	
305	HSIL	POSC	HPV53REF mapping	6680	SNV	T	A	94429	94573	99.85	37.14	HPV53-L1:c.1023T>A	
305	HSIL	POSC	HPV53REF mapping	6686	SNV	C	T	96952	97167	99.78	37.30	HPV53-L1:c.1029C>T	

305	HSIL	POSC	HPV53REF mapping	6707	SNV	T	C	97200	97674	99.51	36.93	HPV53-L1:c.1050T>C	
305	HSIL	POSC	HPV53REF mapping	6743	SNV	A	G	96008	96097	99.91	37.00	HPV53-L1:c.1086A>G	
305	HSIL	POSC	HPV53REF mapping	6911	SNV	G	A	76474	79230	96.52	37.33	HPV53-L1:c.1254G>A	
305	HSIL	POSC	HPV53REF mapping	6911	Deletion	G	-	2604	79230	3.29	35.05	HPV53-L1:c.1254delG	HPV53-L1:p.Ser420fs
305	HSIL	POSC	HPV53REF mapping	7001	SNV	G	A	37238	37296	99.84	37.30	HPV53-L1:c.1344G>A	
305	HSIL	POSC	HPV53REF mapping	7033	SNV	T	A	8608	23252	37.02	36.30	HPV53-L1:c.1376T>A	HPV53-L1:p.Phe459Tyr
305	HSIL	POSC	HPV53REF mapping	7037	SNV	T	C	2627	5971	44.00	35.18	HPV53-L1:c.1380T>C	
305	HSIL	POSC	HPV53REF mapping	7037	Deletion	T	-	272	5971	4.56	34.79	HPV53-L1:c.1380delT	HPV53-L1:p.Gly462fs
305	HSIL	POSC	HPV53REF mapping	7040	SNV	T	G	23	707	3.25	35.91	HPV53-L1:c.1383T>G	
305	HSIL	POSC	HPV66REF mapping	6597	SNV	C	A	54	166	32.53	35.39	HPV66-L1:c.951C>A	
305	HSIL	POSC	HPV66REF mapping	6597	SNV	C	T	111	166	66.87	35.56	HPV66-L1:c.951C>T	
305	HSIL	POSC	HPV66REF mapping	6603	SNV	T	C	171	526	32.51	36.21	HPV66-L1:c.957T>C	
305	HSIL	POSC	HPV66REF mapping	6631	SNV	T	C	49	929	5.27	36.39	HPV66-L1:c.985T>C	HPV66-L1:p.Phe329Leu
305	HSIL	POSC	HPV66REF mapping	6660	SNV	C	T	1059	1062	99.72	37.23	HPV66-L1:c.1014C>T	
305	HSIL	POSC	HPV66REF mapping	6680	SNV	C	T	13	1159	1.12	37.77	HPV66-L1:c.1034C>T	HPV66-L1:p.Ala345Val
305	HSIL	POSC	HPV66REF mapping	6686	SNV	A	C	1152	1155	99.74	36.96	HPV66-L1:c.1040A>C	HPV66-L1:p.Lys347Thr
305	HSIL	POSC	HPV66REF mapping	6692	SNV	C	T	29	1201	2.41	36.10	HPV66-L1:c.1046C>T	HPV66-L1:p.Thr349Ile
305	HSIL	POSC	HPV66REF mapping	6697	SNV	A	G	35	1263	2.77	37.31	HPV66-L1:c.1051A>G	HPV66-L1:p.Thr351Ala
305	HSIL	POSC	HPV66REF mapping	6711	SNV	C	A	1154	1155	99.91	37.22	HPV66-L1:c.1065C>A	
305	HSIL	POSC	HPV66REF mapping	6750	SNV	T	C	57	1184	4.81	36.65	HPV66-L1:c.1104T>C	
305	HSIL	POSC	HPV66REF mapping	6799	SNV	G	A	16	996	1.61	36.19	HPV66-L1:c.1153G>A	HPV66-L1:p.Val385Ile
305	HSIL	POSC	HPV66REF mapping	6849	SNV	T	C	946	947	99.89	37.34	HPV66-L1:c.1203T>C	
305	HSIL	POSC	HPV66REF mapping	6855	SNV	C	A	1072	1074	99.81	36.73	HPV66-L1:c.1209C>A	
305	HSIL	POSC	HPV66REF mapping	6900	SNV	T	C	53	1081	4.90	36.49	HPV66-L1:c.1254T>C	
305	HSIL	POSC	HPV66REF mapping	6927	SNV	G	A	1080	1086	99.45	37.23	HPV66-L1:c.1281G>A	
305	HSIL	POSC	HPV66REF mapping	6960	SNV	C	A	15	1043	1.44	37.87	HPV66-L1:c.1314C>A	
305	HSIL	POSC	HPV66REF mapping	6975	SNV	G	A	13	975	1.33	35.62	HPV66-L1:c.1329G>A	
305	HSIL	POSC	HPV66REF mapping	6984	SNV	A	G	804	809	99.38	37.04	HPV66-L1:c.1338A>G	
305	HSIL	POSC	HPV66REF mapping	6987	SNV	T	C	12	828	1.45	37.92	HPV66-L1:c.1341T>C	
305	HSIL	POSC	HPV66REF mapping	6996	MNV	GG	AA	10	761	1.31	37.00	HPV66-L1:c.1350_1351delGGinsAA	HPV66-L1:p.Asp451Asn
305	HSIL	POSC	HPV66REF mapping	7002	SNV	C	T	9	673	1.34	37.89	HPV66-L1:c.1356C>T	
305	HSIL	POSC	HPV66REF mapping	7011	SNV	A	T	7	572	1.22	34.14	HPV66-L1:c.1365A>T	
305	HSIL	POSC	HPV66REF mapping	7014	MNV	CC	TT	6	569	1.05	34.60	HPV66-L1:c.1368_1369delCCinsTT	
305	HSIL	POSC	HPV66REF mapping	7017	SNV	G	A	543	552	98.37	36.64	HPV66-L1:c.1371G>A	
305	HSIL	POSC	HPV66REF mapping	7022	SNV	A	C	6	399	1.50	38.00	HPV66-L1:c.1376A>C	HPV66-L1:p.Gln459Pro
305	HSIL	POSC	HPV66REF mapping	7025	SNV	T	A	172	352	48.86	35.78	HPV66-L1:c.1379T>A	HPV66-L1:p.Phe460Tyr
305	HSIL	POSC	HPV70REF mapping	6537	SNV	A	T	94	136	69.12	35.23	HPV70-L1:c.948A>T	
305	HSIL	POSC	HPV70REF mapping	6537	Deletion	A	-	3	136	2.21	33.33	HPV70-L1:c.948delA	HPV70-L1:p.His317fs
305	HSIL	POSC	HPV70REF mapping	6540	SNV	C	T	219	220	99.55	36.52	HPV70-L1:c.951C>T	
305	HSIL	POSC	HPV70REF mapping	6543	SNV	T	C	142	365	38.90	36.05	HPV70-L1:c.954T>C	
305	HSIL	POSC	HPV70REF mapping	6552	SNV	T	C	6	396	1.52	33.83	HPV70-L1:c.963T>C	
305	HSIL	POSC	HPV70REF mapping	6602	SNV	C	T	155	832	18.63	36.72	HPV70-L1:c.1013C>T	HPV70-L1:p.Thr338Ile
305	HSIL	POSC	HPV70REF mapping	6633	SNV	G	A	836	838	99.76	37.33	HPV70-L1:c.1044G>A	
305	HSIL	POSC	HPV70REF mapping	6683	SNV	A	G	422	698	60.46	37.07	HPV70-L1:c.1094A>G	HPV70-L1:p.His365Arg
305	HSIL	POSC	HPV70REF mapping	6693	SNV	A	G	7	627	1.12	36.71	HPV70-L1:c.1104A>G	
305	HSIL	POSC	HPV70REF mapping	6704	SNV	A	G	103	554	18.59	37.06	HPV70-L1:c.1115A>G	HPV70-L1:p.Gln372Arg
305	HSIL	POSC	HPV70REF mapping	6741	SNV	T	A	636	640	99.38	37.41	HPV70-L1:c.1152T>A	
305	HSIL	POSC	HPV70REF mapping	6757	SNV	A	G	143	761	18.79	36.53	HPV70-L1:c.1168A>G	HPV70-L1:p.Ile390Val
305	HSIL	POSC	HPV70REF mapping	6801	SNV	A	C	689	690	99.86	36.85	HPV70-L1:c.1212A>C	

305	HSIL	POSC	HPV70REF mapping	6808	SNV	C	A	10	749	1.34	36.50	HPV70-L1:c.1219C>A	HPV70-L1:p.Pro407Thr
305	HSIL	POSC	HPV70REF mapping	6872	MNV	AA	TG	93	640	14.53	36.91	HPV70-L1:c.1283_1284delAAinsTG	HPV70-L1:p.Gln428Leu
305	HSIL	POSC	HPV70REF mapping	6873	SNV	A	G	540	637	84.77	36.89	HPV70-L1:c.1284A>G	
305	HSIL	POSC	HPV70REF mapping	6886	SNV	A	G	590	591	99.83	36.78	HPV70-L1:c.1297A>G	HPV70-L1:p.Thr433Ala
305	HSIL	POSC	HPV70REF mapping	6893	Deletion	A	-	7	557	1.26	37.43	HPV70-L1:c.1310delA	HPV70-L1:p.Lys437fs
305	HSIL	POSC	HPV70REF mapping	6906	SNV	C	A	7	580	1.21	38.00	HPV70-L1:c.1317C>A	
305	HSIL	POSC	HPV70REF mapping	6968	SNV	A	C	4	171	2.34	38.00	HPV70-L1:c.1379A>C	HPV70-L1:p.Gln460Pro
305	HSIL	POSC	HPV70REF mapping	6968	SNV	A	T	2	171	1.17	36.00	HPV70-L1:c.1379A>T	HPV70-L1:p.Gln460Leu
305	HSIL	POSC	HPV70REF mapping	6971	SNV	T	A	73	149	48.99	36.77	HPV70-L1:c.1382T>A	HPV70-L1:p.Phe461Tyr
313	HSIL	CARC	HPV16REF mapping	6592	SNV	C	A	8235	20798	39.60	35.29	HPV16-L1:c.954C>A	
313	HSIL	CARC	HPV16REF mapping	6592	SNV	C	T	12422	20798	59.73	35.30	HPV16-L1:c.954C>T	
313	HSIL	CARC	HPV16REF mapping	6595	SNV	C	T	31096	31157	99.80	36.80	HPV16-L1:c.957C>T	
313	HSIL	CARC	HPV16REF mapping	6598	SNV	T	C	8647	51474	16.80	36.47	HPV16-L1:c.960T>C	
313	HSIL	CARC	HPV16REF mapping	7023	SNV	T	A	10921	26499	41.21	36.08	HPV16-L1:c.1385T>A	HPV16-L1:p.Phe462Tyr
313	HSIL	CARC	HPV16REF mapping	7027	SNV	T	C	295	1452	20.32	35.61	HPV16-L1:c.1389T>C	
319	HSIL	CARC	HPV16REF mapping	6592	SNV	C	A	9649	21342	45.21	35.23	HPV16-L1:c.954C>A	
319	HSIL	CARC	HPV16REF mapping	6592	SNV	C	T	11558	21342	54.16	35.10	HPV16-L1:c.954C>T	
319	HSIL	CARC	HPV16REF mapping	6595	SNV	C	T	31770	31851	99.75	36.70	HPV16-L1:c.957C>T	
319	HSIL	CARC	HPV16REF mapping	6598	SNV	T	C	5493	54409	10.10	36.35	HPV16-L1:c.960T>C	
319	HSIL	CARC	HPV16REF mapping	7020	SNV	A	C	390	18952	2.06	37.12	HPV16-L1:c.1382A>C	HPV16-L1:p.Gln461Pro
319	HSIL	CARC	HPV16REF mapping	7023	SNV	T	A	5116	16872	30.32	35.84	HPV16-L1:c.1385T>A	HPV16-L1:p.Phe462Tyr
319	HSIL	CARC	HPV16REF mapping	7027	SNV	T	C	539	2954	18.25	35.16	HPV16-L1:c.1389T>C	
319	HSIL	POSC	HPV70REF mapping	6537	SNV	A	T	608	919	66.16	35.08	HPV70-L1:c.948A>T	
319	HSIL	POSC	HPV70REF mapping	6537	Deletion	A	-	11	919	1.20	36.45	HPV70-L1:c.948delA	HPV70-L1:p.His317fs
319	HSIL	POSC	HPV70REF mapping	6540	SNV	C	T	1404	1410	99.57	36.55	HPV70-L1:c.951C>T	
319	HSIL	POSC	HPV70REF mapping	6543	SNV	T	C	1125	2382	47.23	36.43	HPV70-L1:c.954T>C	
319	HSIL	POSC	HPV70REF mapping	6560	SNV	A	G	785	3372	23.28	36.81	HPV70-L1:c.971A>G	HPV70-L1:p.His324Arg
319	HSIL	POSC	HPV70REF mapping	6630	SNV	A	G	134	5692	2.35	36.22	HPV70-L1:c.1041A>G	
319	HSIL	POSC	HPV70REF mapping	6633	SNV	G	A	5520	5609	98.41	37.06	HPV70-L1:c.1044G>A	
319	HSIL	POSC	HPV70REF mapping	6637	SNV	A	G	96	5796	1.66	36.55	HPV70-L1:c.1048A>G	HPV70-L1:p.Ile350Val
319	HSIL	POSC	HPV70REF mapping	6642	SNV	T	C	232	5747	4.04	36.45	HPV70-L1:c.1053T>C	
319	HSIL	POSC	HPV70REF mapping	6643	SNV	G	T	75	5949	1.26	36.28	HPV70-L1:c.1054G>T	HPV70-L1:p.Ala352Ser
319	HSIL	POSC	HPV70REF mapping	6655	SNV	C	T	145	5132	2.83	37.01	HPV70-L1:c.1066C>T	HPV70-L1:p.Pro356Ser
319	HSIL	POSC	HPV70REF mapping	6671	SNV	A	G	59	4950	1.19	36.83	HPV70-L1:c.1082A>G	HPV70-L1:p.Glu361Gly
319	HSIL	POSC	HPV70REF mapping	6678	SNV	T	C	71	4919	1.44	36.04	HPV70-L1:c.1089T>C	
319	HSIL	POSC	HPV70REF mapping	6679	SNV	A	G	167	4946	3.38	35.80	HPV70-L1:c.1090A>G	HPV70-L1:p.Arg364Gly
319	HSIL	POSC	HPV70REF mapping	6683	SNV	A	G	755	4673	16.16	36.73	HPV70-L1:c.1094A>G	HPV70-L1:p.His365Arg
319	HSIL	POSC	HPV70REF mapping	6694	SNV	T	C	52	4177	1.24	36.90	HPV70-L1:c.1105T>C	HPV70-L1:p.Tyr369His
319	HSIL	POSC	HPV70REF mapping	6741	SNV	T	A	4531	4544	99.71	37.13	HPV70-L1:c.1152T>A	
319	HSIL	POSC	HPV70REF mapping	6757	SNV	A	G	129	5335	2.42	35.97	HPV70-L1:c.1168A>G	HPV70-L1:p.Ile390Val
319	HSIL	POSC	HPV70REF mapping	6765	SNV	T	C	199	5221	3.81	36.18	HPV70-L1:c.1176T>C	
319	HSIL	POSC	HPV70REF mapping	6771	SNV	T	C	162	4983	3.25	36.30	HPV70-L1:c.1182T>C	
319	HSIL	POSC	HPV70REF mapping	6799	SNV	G	A	115	5333	2.16	36.74	HPV70-L1:c.1210G>A	HPV70-L1:p.Gly404Arg
319	HSIL	POSC	HPV70REF mapping	6801	SNV	A	C	4826	4838	99.75	36.96	HPV70-L1:c.1212A>C	
319	HSIL	POSC	HPV70REF mapping	6823	SNV	A	G	101	4921	2.05	37.10	HPV70-L1:c.1234A>G	HPV70-L1:p.Ser412Gly
319	HSIL	POSC	HPV70REF mapping	6844	SNV	T	C	96	5583	1.72	36.25	HPV70-L1:c.1255T>C	HPV70-L1:p.Tyr419His
319	HSIL	POSC	HPV70REF mapping	6845	SNV	A	G	90	5042	1.79	36.88	HPV70-L1:c.1256A>G	HPV70-L1:p.Tyr419Cys
319	HSIL	POSC	HPV70REF mapping	6854	SNV	C	T	118	4427	2.67	36.90	HPV70-L1:c.1265C>T	HPV70-L1:p.Ser422Leu
319	HSIL	POSC	HPV70REF mapping	6861	SNV	T	C	57	4783	1.19	36.67	HPV70-L1:c.1272T>C	

319	HSIL	POSC	HPV70REF mapping	6864	SNV	A	G	114	4836	2.36	36.90	HPV70-L1:c.1275A>G	HPV70-L1:p.Ile425Met
319	HSIL	POSC	HPV70REF mapping	6867	SNV	A	G	141	4621	3.05	36.82	HPV70-L1:c.1278A>G	
319	HSIL	POSC	HPV70REF mapping	6873	SNV	A	G	4434	4481	98.95	36.99	HPV70-L1:c.1284A>G	
319	HSIL	POSC	HPV70REF mapping	6882	SNV	T	C	68	4448	1.53	35.99	HPV70-L1:c.1293T>C	
319	HSIL	POSC	HPV70REF mapping	6886	SNV	A	G	4334	4337	99.93	36.73	HPV70-L1:c.1297A>G	HPV70-L1:p.Thr433Ala
319	HSIL	POSC	HPV70REF mapping	6893	Deletion	A	-	151	4101	3.68	36.42	HPV70-L1:c.1310delA	HPV70-L1:p.Lys437fs
319	HSIL	POSC	HPV70REF mapping	6902	SNV	A	G	65	4000	1.63	35.32	HPV70-L1:c.1313A>G	HPV70-L1:p.Asp438Gly
319	HSIL	POSC	HPV70REF mapping	6921	SNV	A	G	82	3673	2.23	36.99	HPV70-L1:c.1332A>G	
319	HSIL	POSC	HPV70REF mapping	6936	SNV	T	C	38	3441	1.10	36.82	HPV70-L1:c.1347T>C	
319	HSIL	POSC	HPV70REF mapping	6968	SNV	A	C	22	1311	1.68	36.55	HPV70-L1:c.1379A>C	HPV70-L1:p.Gln460Pro
319	HSIL	POSC	HPV70REF mapping	6971	SNV	T	A	610	1122	54.37	36.20	HPV70-L1:c.1382T>A	HPV70-L1:p.Phe461Tyr
319	HSIL	POSC	HPV70REF mapping	6975	SNV	T	C	15	131	11.45	36.33	HPV70-L1:c.1386T>C	
386	HSIL	CARC	HPV16REF mapping	6598	SNV	T	C	10	157	6.37	36.50	HPV16-L1:c.960T>C	
386	HSIL	CARC	HPV16REF mapping	6614	SNV	G	T	3	205	1.46	29.67	HPV16-L1:c.976G>T	HPV16-L1:p.Gly326Cys
386	HSIL	CARC	HPV16REF mapping	6732	SNV	G	T	6	476	1.26	35.50	HPV16-L1:c.1094G>T	HPV16-L1:p.Arg365Leu
386	HSIL	CARC	HPV16REF mapping	6771	SNV	T	C	11	504	2.18	36.91	HPV16-L1:c.1133T>C	HPV16-L1:p.Leu378Pro
386	HSIL	CARC	HPV16REF mapping	6773	SNV	T	C	9	519	1.73	34.67	HPV16-L1:c.1135T>C	HPV16-L1:p.Cys379Arg
386	HSIL	CARC	HPV16REF mapping	6854	SNV	C	T	7	544	1.29	35.71	HPV16-L1:c.1216C>T	
386	HSIL	CARC	HPV16REF mapping	6923	SNV	C	A	5	421	1.19	38.00	HPV16-L1:c.1285C>A	HPV16-L1:p.Gln429Lys
386	HSIL	CARC	HPV16REF mapping	6923	SNV	C	T	5	421	1.19	36.20	HPV16-L1:c.1285C>T	HPV16-L1:p.Gln429*
386	HSIL	CARC	HPV16REF mapping	7023	SNV	T	A	68	110	61.82	36.40	HPV16-L1:c.1385T>A	HPV16-L1:p.Phe462Tyr
386	HSIL	NC	HPV83REF mapping	6697	SNV	A	T	124	161	77.02	37.31	HPV83-L1:c.956A>T	HPV83-L1:p.Gln319Leu
386	HSIL	NC	HPV83REF mapping	6701	SNV	A	T	3134	9672	32.40	35.23	HPV83-L1:c.960A>T	
386	HSIL	NC	HPV83REF mapping	6701	Deletion	A	-	115	9672	1.19	35.57	HPV83-L1:c.960delA	HPV83-L1:p.His321fs
386	HSIL	NC	HPV83REF mapping	6707	SNV	T	C	5546	26198	21.17	36.57	HPV83-L1:c.966T>C	
386	HSIL	NC	HPV83REF mapping	6845	SNV	A	C	71470	71787	99.56	36.92	HPV83-L1:c.1104A>C	
386	HSIL	NC	HPV83REF mapping	6893	SNV	G	T	67896	67952	99.92	37.06	HPV83-L1:c.1152G>T	
386	HSIL	NC	HPV83REF mapping	6941	SNV	A	G	59241	59528	99.52	37.03	HPV83-L1:c.1200A>G	
386	HSIL	NC	HPV83REF mapping	6968	SNV	G	A	61767	61899	99.79	36.71	HPV83-L1:c.1227G>A	
386	HSIL	NC	HPV83REF mapping	6974	SNV	C	T	62450	62797	99.45	37.03	HPV83-L1:c.1233C>T	
386	HSIL	NC	HPV83REF mapping	7124	SNV	A	G	17022	17073	99.70	36.56	HPV83-L1:c.1383A>G	
386	HSIL	NC	HPV83REF mapping	7132	SNV	T	A	5888	12729	46.26	36.46	HPV83-L1:c.1391T>A	HPV83-L1:p.Phe464Tyr
386	HSIL	NC	HPV83REF mapping	7136	SNV	T	C	159	606	26.24	35.31	HPV83-L1:c.1395T>C	
386	HSIL	NC	HPV83REF mapping	7137	Insertion	-	C	6	264	2.27	35.17	HPV83-L1:c.1395_1396insC	HPV83-L1:p.Arg468fs
386	HSIL	NC	HPV83REF mapping	7137	SNV	T	C	31	264	11.74	35.45	HPV83-L1:c.1396T>C	
399	HSIL	CARC	HPV16REF mapping	6592	SNV	C	A	4005	10624	37.70	35.20	HPV16-L1:c.954C>A	
399	HSIL	CARC	HPV16REF mapping	6592	SNV	C	T	6518	10624	61.35	35.20	HPV16-L1:c.954C>T	
399	HSIL	CARC	HPV16REF mapping	6595	SNV	C	T	16561	16606	99.73	36.71	HPV16-L1:c.957C>T	
399	HSIL	CARC	HPV16REF mapping	6598	SNV	T	C	5762	28442	20.26	36.31	HPV16-L1:c.960T>C	
399	HSIL	CARC	HPV16REF mapping	6612	SNV	G	T	632	35650	1.77	37.15	HPV16-L1:c.974G>T	HPV16-L1:p.Trp325Leu
399	HSIL	CARC	HPV16REF mapping	6613	SNV	G	T	898	36200	2.48	37.11	HPV16-L1:c.975G>T	HPV16-L1:p.Trp325Cys
399	HSIL	CARC	HPV16REF mapping	6737	SNV	G	T	1132	60709	1.86	37.00	HPV16-L1:c.1099G>T	HPV16-L1:p.Gly367Trp
399	HSIL	CARC	HPV16REF mapping	6738	SNV	G	T	927	61143	1.52	37.07	HPV16-L1:c.1100G>T	HPV16-L1:p.Gly367Val
399	HSIL	CARC	HPV16REF mapping	6865	SNV	C	A	1118	60387	1.85	36.82	HPV16-L1:c.1227C>A	
399	HSIL	CARC	HPV16REF mapping	6866	SNV	C	A	967	60464	1.60	37.11	HPV16-L1:c.1228C>A	HPV16-L1:p.Pro410Thr
399	HSIL	CARC	HPV16REF mapping	6867	SNV	C	A	784	60427	1.30	36.96	HPV16-L1:c.1229C>A	HPV16-L1:p.Pro410Gln
399	HSIL	CARC	HPV16REF mapping	6908	SNV	C	A	740	48269	1.53	36.91	HPV16-L1:c.1270C>A	HPV16-L1:p.Gln424Lys
399	HSIL	CARC	HPV16REF mapping	6958	SNV	C	A	493	34641	1.42	36.75	HPV16-L1:c.1320C>A	
399	HSIL	CARC	HPV16REF mapping	6959	SNV	C	A	657	34101	1.93	37.00	HPV16-L1:c.1321C>A	HPV16-L1:p.Leu441Ile

399	HSIL	CARC	HPV16REF mapping	6978	SNV	G	T	529	31069	1.70	37.05	HPV16-L1:c.1340G>T	HPV16-L1:p.Trp447Leu
399	HSIL	CARC	HPV16REF mapping	7020	SNV	A	C	223	14233	1.57	36.93	HPV16-L1:c.1382A>C	HPV16-L1:p.Gln461Pro
399	HSIL	CARC	HPV16REF mapping	7023	SNV	T	A	5259	12806	41.07	35.91	HPV16-L1:c.1385T>A	HPV16-L1:p.Phe462Tyr
399	HSIL	CARC	HPV16REF mapping	7027	SNV	T	C	126	995	12.66	35.52	HPV16-L1:c.1389T>C	
399	HSIL	CARC	HPV16REF mapping	7027	MNV	TT	CC	25	996	2.51	37.50	HPV16-L1:c.1389_1390delTTinsCC	
399	HSIL	CARC	HPV16REF mapping	7028	SNV	T	C	6	274	2.19	37.67	HPV16-L1:c.1390T>C	
81	LSIL	CARC	HPV58REF mapping	6596	SNV	T	A	1022	5860	17.44	35.10	HPV58-L1:c.954T>A	
81	LSIL	CARC	HPV58REF mapping	6602	SNV	C	T	1903	11243	16.93	36.52	HPV58-L1:c.960C>T	
81	LSIL	CARC	HPV58REF mapping	6617	SNV	G	T	184	13425	1.37	37.02	HPV58-L1:c.975G>T	HPV58-L1:p.Trp325Cys
81	LSIL	CARC	HPV58REF mapping	6692	SNV	G	A	14491	14580	99.39	36.70	HPV58-L1:c.1050G>A	
81	LSIL	CARC	HPV58REF mapping	6697	SNV	G	A	12847	12898	99.60	36.59	HPV58-L1:c.1055G>A	HPV58-L1:p.Gly352Asp
81	LSIL	CARC	HPV58REF mapping	6711	SNV	G	A	11881	11895	99.88	37.17	HPV58-L1:c.1069G>A	HPV58-L1:p.Asp357Asn
81	LSIL	CARC	HPV58REF mapping	6750	SNV	G	T	412	22475	1.83	37.25	HPV58-L1:c.1108G>T	HPV58-L1:p.Asp370Tyr
81	LSIL	CARC	HPV58REF mapping	6798	SNV	A	G	21581	21596	99.93	36.71	HPV58-L1:c.1156A>G	HPV58-L1:p.Ile386Val
81	LSIL	CARC	HPV58REF mapping	6822	SNV	G	A	21801	21888	99.60	36.72	HPV58-L1:c.1180G>A	HPV58-L1:p.Asp394Asn
81	LSIL	CARC	HPV58REF mapping	6827	MNV	CA	AG	21550	21589	99.82	36.73	HPV58-L1:c.1185_1186delCAinsAG	HPV58-L1:p.Asn396Asp
81	LSIL	CARC	HPV58REF mapping	6909	SNV	C	A	387	21781	1.78	37.10	HPV58-L1:c.1267C>A	HPV58-L1:p.Gln423Lys
81	LSIL	CARC	HPV58REF mapping	6938	SNV	C	A	321	19197	1.67	36.50	HPV58-L1:c.1296C>A	
81	LSIL	CARC	HPV58REF mapping	6939	SNV	C	A	480	19143	2.51	36.58	HPV58-L1:c.1297C>A	HPV58-L1:p.Pro433Thr
81	LSIL	CARC	HPV58REF mapping	6940	SNV	C	A	312	19134	1.63	36.81	HPV58-L1:c.1298C>A	HPV58-L1:p.Pro433His
81	LSIL	CARC	HPV58REF mapping	6979	SNV	G	T	218	11742	1.86	36.81	HPV58-L1:c.1337G>T	HPV58-L1:p.Trp446Leu
81	LSIL	CARC	HPV58REF mapping	7016	SNV	A	G	7306	7362	99.24	36.60	HPV58-L1:c.1374A>G	
81	LSIL	CARC	HPV58REF mapping	7020	SNV	C	A	91	6355	1.43	36.88	HPV58-L1:c.1378C>A	HPV58-L1:p.Gln460Lys
81	LSIL	CARC	HPV58REF mapping	7024	SNV	T	A	2511	5418	46.35	36.18	HPV58-L1:c.1382T>A	HPV58-L1:p.Phe461Tyr
81	LSIL	CARC	HPV58REF mapping	7028	SNV	T	C	44	268	16.42	34.00	HPV58-L1:c.1386T>C	
81	LSIL	CARC	HPV58REF mapping	7028	MNV	TT	CC	7	268	2.61	35.95	HPV58-L1:c.1386_1387delTTinsCC	
81	LSIL	NC	HPV61REF mapping	6720	SNV	C	A	434	1070	40.56	35.10	HPV61-L1:c.957C>A	
81	LSIL	NC	HPV61REF mapping	6720	SNV	C	T	611	1070	57.10	35.19	HPV61-L1:c.957C>T	
81	LSIL	NC	HPV61REF mapping	6720	Deletion	C	-	22	1070	2.06	37.00	HPV61-L1:c.958delC	HPV61-L1:p.His320fs
81	LSIL	NC	HPV61REF mapping	6723	SNV	C	T	1402	1407	99.64	36.88	HPV61-L1:c.960C>T	
81	LSIL	NC	HPV61REF mapping	6726	SNV	C	T	173	2895	5.98	35.98	HPV61-L1:c.963C>T	
81	LSIL	NC	HPV61REF mapping	6778	SNV	C	A	92	5155	1.78	37.18	HPV61-L1:c.1015C>A	HPV61-L1:p.Arg339Ser
81	LSIL	NC	HPV61REF mapping	6814	SNV	C	A	94	6261	1.50	36.86	HPV61-L1:c.1051C>A	HPV61-L1:p.Pro351Thr
81	LSIL	NC	HPV61REF mapping	6816	SNV	C	A	85	6534	1.30	37.07	HPV61-L1:c.1053C>A	
81	LSIL	NC	HPV61REF mapping	6817	SNV	C	A	107	6531	1.64	36.59	HPV61-L1:c.1054C>A	HPV61-L1:p.Pro352Thr
81	LSIL	NC	HPV61REF mapping	6818	SNV	C	A	92	6660	1.38	37.14	HPV61-L1:c.1055C>A	HPV61-L1:p.Pro352His
81	LSIL	NC	HPV61REF mapping	6919	SNV	C	A	110	6074	1.81	36.64	HPV61-L1:c.1156C>A	HPV61-L1:p.Pro386Thr
81	LSIL	NC	HPV61REF mapping	6967	SNV	G	T	124	7195	1.72	37.62	HPV61-L1:c.1204G>T	HPV61-L1:p.Asp402Tyr
81	LSIL	NC	HPV61REF mapping	6996	SNV	C	A	150	7744	1.94	36.97	HPV61-L1:c.1233C>A	
81	LSIL	NC	HPV61REF mapping	7056	SNV	G	T	113	6474	1.75	36.97	HPV61-L1:c.1293G>T	HPV61-L1:p.Lys431Asn
81	LSIL	NC	HPV61REF mapping	7070	SNV	C	A	72	5515	1.31	37.26	HPV61-L1:c.1307C>A	HPV61-L1:p.Pro436Gln
81	LSIL	NC	HPV61REF mapping	7077	SNV	C	A	107	5765	1.86	36.87	HPV61-L1:c.1314C>A	
81	LSIL	NC	HPV61REF mapping	7154	SNV	T	A	758	1558	48.65	36.28	HPV61-L1:c.1391T>A	HPV61-L1:p.Phe464Tyr
81	LSIL	NC	HPV61REF mapping	7158	SNV	T	C	24	107	22.43	35.67	HPV61-L1:c.1395T>C	
137	LSIL	POSC	HPV66REF mapping	6597	SNV	C	A	5743	17017	33.75	35.21	HPV66-L1:c.951C>A	
137	LSIL	POSC	HPV66REF mapping	6597	SNV	C	T	11035	17017	64.85	35.25	HPV66-L1:c.951C>T	
137	LSIL	POSC	HPV66REF mapping	6603	SNV	T	C	10030	50716	19.78	36.24	HPV66-L1:c.957T>C	
137	LSIL	POSC	HPV66REF mapping	6617	SNV	G	T	1163	79316	1.47	37.17	HPV66-L1:c.971G>T	HPV66-L1:p.Trp324Leu
137	LSIL	POSC	HPV66REF mapping	6618	SNV	G	T	1795	79677	2.25	37.34	HPV66-L1:c.972G>T	HPV66-L1:p.Trp324Cys

137	LSIL	POSC	HPV66REF mapping	6862	SNV	C	A	1255	94707	1.33	37.05	HPV66-L1:c.1216C>A	HPV66-L1:p.Pro406Thr
137	LSIL	POSC	HPV66REF mapping	6863	SNV	C	A	1213	95898	1.26	36.98	HPV66-L1:c.1217C>A	HPV66-L1:p.Pro406Gln
137	LSIL	POSC	HPV66REF mapping	6929	SNV	G	T	1290	95776	1.35	37.19	HPV66-L1:c.1283G>T	HPV66-L1:p.Arg428Met
137	LSIL	POSC	HPV66REF mapping	6940	SNV	C	A	1251	92524	1.35	37.30	HPV66-L1:c.1294C>A	HPV66-L1:p.Pro432Thr
137	LSIL	POSC	HPV66REF mapping	6941	SNV	C	A	1181	93991	1.26	37.33	HPV66-L1:c.1295C>A	HPV66-L1:p.Pro432His
137	LSIL	POSC	HPV66REF mapping	6960	SNV	C	A	1624	88623	1.83	37.00	HPV66-L1:c.1314C>A	
137	LSIL	POSC	HPV66REF mapping	6961	SNV	C	A	1306	88449	1.48	37.15	HPV66-L1:c.1315C>A	HPV66-L1:p.Leu439Met
137	LSIL	POSC	HPV66REF mapping	6980	SNV	G	T	1012	74211	1.36	37.05	HPV66-L1:c.1334G>T	HPV66-L1:p.Trp445Leu
137	LSIL	POSC	HPV66REF mapping	7025	SNV	T	A	12539	28922	43.35	36.22	HPV66-L1:c.1379T>A	HPV66-L1:p.Phe460Tyr
137	LSIL	POSC	HPV66REF mapping	7029	SNV	T	C	355	2227	15.94	35.50	HPV66-L1:c.1383T>C	
137	LSIL	POSC	HPV66REF mapping	7030	Insertion	-	C	18	795	2.26	35.39	HPV66-L1:c.1383_1384insC	HPV66-L1:p.Arg464fs
137	LSIL	POSC	HPV66REF mapping	7030	SNV	T	C	116	794	14.61	34.89	HPV66-L1:c.1384T>C	
138	LSIL	NC	HPV81REF mapping	6817	SNV	C	A	4349	12838	33.88	35.57	HPV81-L1:c.960C>A	
138	LSIL	NC	HPV81REF mapping	6817	SNV	C	T	8374	12838	65.23	35.43	HPV81-L1:c.960C>T	
138	LSIL	NC	HPV81REF mapping	6823	SNV	T	C	5089	31904	15.95	36.41	HPV81-L1:c.966T>C	
138	LSIL	NC	HPV81REF mapping	6827	SNV	G	T	538	33092	1.63	37.39	HPV81-L1:c.970G>T	HPV81-L1:p.Gly324Cys
138	LSIL	NC	HPV81REF mapping	6845	SNV	G	T	769	40755	1.89	37.09	HPV81-L1:c.988G>T	HPV81-L1:p.Glu330*
138	LSIL	NC	HPV81REF mapping	6848	SNV	A	C	40797	40824	99.93	37.25	HPV81-L1:c.991A>C	HPV81-L1:p.Met331Leu
138	LSIL	NC	HPV81REF mapping	6956	SNV	C	A	1033	70883	1.46	37.17	HPV81-L1:c.1099C>A	HPV81-L1:p.His367Asn
138	LSIL	NC	HPV81REF mapping	7042	SNV	G	T	1181	63822	1.85	36.99	HPV81-L1:c.1185G>T	HPV81-L1:p.Met395Ile
138	LSIL	NC	HPV81REF mapping	7084	SNV	A	G	57529	57707	99.69	36.55	HPV81-L1:c.1227A>G	
138	LSIL	NC	HPV81REF mapping	7106	SNV	G	T	1149	61339	1.87	36.77	HPV81-L1:c.1249G>T	HPV81-L1:p.Asp417Tyr
138	LSIL	NC	HPV81REF mapping	7150	SNV	G	T	1221	53600	2.28	37.20	HPV81-L1:c.1293G>T	HPV81-L1:p.Lys431Asn
138	LSIL	NC	HPV81REF mapping	7163	SNV	C	A	897	48861	1.84	37.14	HPV81-L1:c.1306C>A	HPV81-L1:p.Pro436Thr
138	LSIL	NC	HPV81REF mapping	7171	SNV	C	A	1166	48362	2.41	36.87	HPV81-L1:c.1314C>A	
138	LSIL	NC	HPV81REF mapping	7182	SNV	C	A	1157	49311	2.35	37.00	HPV81-L1:c.1325C>A	HPV81-L1:p.Pro442His
138	LSIL	NC	HPV81REF mapping	7197	SNV	C	A	647	40735	1.59	36.96	HPV81-L1:c.1340C>A	HPV81-L1:p.Ser447*
138	LSIL	NC	HPV81REF mapping	7211	SNV	G	T	530	36891	1.44	37.02	HPV81-L1:c.1354G>T	HPV81-L1:p.Asp452Tyr
138	LSIL	NC	HPV81REF mapping	7245	SNV	A	C	167	10950	1.53	36.59	HPV81-L1:c.1388A>C	HPV81-L1:p.Gln463Pro
138	LSIL	NC	HPV81REF mapping	7248	SNV	T	A	3915	10370	37.75	36.36	HPV81-L1:c.1391T>A	HPV81-L1:p.Phe464Tyr
138	LSIL	NC	HPV81REF mapping	7252	SNV	T	C	179	960	18.65	35.48	HPV81-L1:c.1395T>C	
138	LSIL	NC	HPV81REF mapping	7253	Insertion	-	C	5	451	1.11	37.20	HPV81-L1:c.1395_1396insC	HPV81-L1:p.Arg468fs
138	LSIL	NC	HPV81REF mapping	7253	SNV	T	C	60	449	13.36	34.70	HPV81-L1:c.1396T>C	
138	LSIL	NC	HPV83REF mapping	6697	SNV	A	T	16	18	88.89	37.19	HPV83-L1:c.956A>T	HPV83-L1:p.Gln319Leu
138	LSIL	NC	HPV83REF mapping	6701	SNV	A	T	213	887	24.01	35.08	HPV83-L1:c.960A>T	
138	LSIL	NC	HPV83REF mapping	6701	Deletion	A	-	13	887	1.47	37.38	HPV83-L1:c.960delA	HPV83-L1:p.His321fs
138	LSIL	NC	HPV83REF mapping	6707	SNV	T	C	426	2471	17.24	36.65	HPV83-L1:c.966T>C	
138	LSIL	NC	HPV83REF mapping	6711	SNV	G	T	40	2571	1.56	36.80	HPV83-L1:c.970G>T	HPV83-L1:p.Gly324Cys
138	LSIL	NC	HPV83REF mapping	6731	MNV	GT	AC	37	3303	1.12	37.08	HPV83-L1:c.990_991delGTinsAC	
138	LSIL	NC	HPV83REF mapping	6734	SNV	A	G	45	3476	1.29	37.27	HPV83-L1:c.993A>G	
138	LSIL	NC	HPV83REF mapping	6749	SNV	A	G	4439	4495	98.75	36.69	HPV83-L1:c.1008A>G	
138	LSIL	NC	HPV83REF mapping	6759	SNV	C	A	130	4708	2.76	37.01	HPV83-L1:c.1018C>A	HPV83-L1:p.Arg340Ser
138	LSIL	NC	HPV83REF mapping	6855	SNV	G	T	114	6157	1.85	37.42	HPV83-L1:c.1114G>T	HPV83-L1:p.Asp372Tyr
138	LSIL	NC	HPV83REF mapping	6897	SNV	C	A	104	5558	1.87	37.20	HPV83-L1:c.1156C>A	HPV83-L1:p.Pro386Thr
138	LSIL	NC	HPV83REF mapping	6898	SNV	C	A	100	5587	1.79	36.80	HPV83-L1:c.1157C>A	HPV83-L1:p.Pro386His
138	LSIL	NC	HPV83REF mapping	6930	SNV	G	T	85	5116	1.66	37.39	HPV83-L1:c.1189G>T	HPV83-L1:p.Glu397*
138	LSIL	NC	HPV83REF mapping	6932	SNV	A	G	4574	4579	99.89	37.12	HPV83-L1:c.1191A>G	
138	LSIL	NC	HPV83REF mapping	6941	SNV	A	G	4791	4843	98.93	37.02	HPV83-L1:c.1200A>G	
138	LSIL	NC	HPV83REF mapping	6945	SNV	G	T	71	4701	1.51	37.39	HPV83-L1:c.1204G>T	HPV83-L1:p.Glu402*

138	LSIL	NC	HPV83REF mapping	6968	SNV	G	A	70	4521	1.55	36.53	HPV83-L1:c.1227G>A	
138	LSIL	NC	HPV83REF mapping	6968	SNV	G	C	4322	4521	95.60	35.90	HPV83-L1:c.1227G>C	
138	LSIL	NC	HPV83REF mapping	6968	MNV	GC	CA	109	4621	2.36	37.00	HPV83-L1:c.1227_1228delGCinsCA	HPV83-L1:p.Pro410Thr
138	LSIL	NC	HPV83REF mapping	6970	SNV	C	A	72	4686	1.54	36.91	HPV83-L1:c.1229C>A	HPV83-L1:p.Pro410His
138	LSIL	NC	HPV83REF mapping	6974	SNV	C	A	94	4877	1.93	36.89	HPV83-L1:c.1233C>A	
138	LSIL	NC	HPV83REF mapping	7014	SNV	C	A	96	4719	2.03	37.24	HPV83-L1:c.1273C>A	HPV83-L1:p.Arg425Ser
138	LSIL	NC	HPV83REF mapping	7034	SNV	G	T	98	4600	2.13	36.56	HPV83-L1:c.1293G>T	HPV83-L1:p.Lys431Asn
138	LSIL	NC	HPV83REF mapping	7047	SNV	C	A	79	4502	1.75	37.09	HPV83-L1:c.1306C>A	HPV83-L1:p.Pro436Thr
138	LSIL	NC	HPV83REF mapping	7053	SNV	C	A	87	4472	1.95	36.76	HPV83-L1:c.1312C>A	HPV83-L1:p.Pro438Thr
138	LSIL	NC	HPV83REF mapping	7054	SNV	C	A	71	4428	1.60	35.96	HPV83-L1:c.1313C>A	HPV83-L1:p.Pro438His
138	LSIL	NC	HPV83REF mapping	7087	SNV	G	T	71	3435	2.07	36.83	HPV83-L1:c.1346G>T	HPV83-L1:p.Trp449Leu
138	LSIL	NC	HPV83REF mapping	7129	SNV	A	C	30	1021	2.94	37.93	HPV83-L1:c.1388A>C	HPV83-L1:p.Gln463Pro
138	LSIL	NC	HPV83REF mapping	7132	SNV	T	A	474	933	50.80	36.35	HPV83-L1:c.1391T>A	HPV83-L1:p.Phe464Tyr
140	LSIL	NC	HPV61REF mapping	6720	SNV	C	A	1573	4158	37.83	35.04	HPV61-L1:c.957C>A	
140	LSIL	NC	HPV61REF mapping	6720	SNV	C	T	2515	4158	60.49	35.16	HPV61-L1:c.957C>T	
140	LSIL	NC	HPV61REF mapping	6720	Deletion	C	-	57	4158	1.37	36.05	HPV61-L1:c.958delC	HPV61-L1:p.His320fs
140	LSIL	NC	HPV61REF mapping	6723	SNV	C	T	5365	5386	99.61	36.99	HPV61-L1:c.960C>T	
140	LSIL	NC	HPV61REF mapping	6726	SNV	C	T	846	10656	7.94	36.08	HPV61-L1:c.963C>T	
140	LSIL	NC	HPV61REF mapping	6778	SNV	C	A	421	18462	2.28	37.49	HPV61-L1:c.1015C>A	HPV61-L1:p.Arg339Ser
140	LSIL	NC	HPV61REF mapping	6814	SNV	C	A	387	21884	1.77	36.38	HPV61-L1:c.1051C>A	HPV61-L1:p.Pro351Thr
140	LSIL	NC	HPV61REF mapping	6815	SNV	C	A	387	22168	1.75	37.06	HPV61-L1:c.1052C>A	HPV61-L1:p.Pro351His
140	LSIL	NC	HPV61REF mapping	6816	SNV	C	A	344	22838	1.51	36.86	HPV61-L1:c.1053C>A	
140	LSIL	NC	HPV61REF mapping	6817	SNV	C	A	481	22821	2.11	36.99	HPV61-L1:c.1054C>A	HPV61-L1:p.Pro352Thr
140	LSIL	NC	HPV61REF mapping	6818	SNV	C	A	369	23287	1.58	37.09	HPV61-L1:c.1055C>A	HPV61-L1:p.Pro352His
140	LSIL	NC	HPV61REF mapping	6848	SNV	G	T	351	23159	1.52	37.24	HPV61-L1:c.1085G>T	HPV61-L1:p.Arg362Met
140	LSIL	NC	HPV61REF mapping	6919	SNV	C	A	349	19566	1.78	37.16	HPV61-L1:c.1156C>A	HPV61-L1:p.Pro386Thr
140	LSIL	NC	HPV61REF mapping	6920	SNV	C	A	277	19697	1.41	36.97	HPV61-L1:c.1157C>A	HPV61-L1:p.Pro386His
140	LSIL	NC	HPV61REF mapping	6967	SNV	G	T	350	23249	1.51	37.40	HPV61-L1:c.1204G>T	HPV61-L1:p.Asp402Tyr
140	LSIL	NC	HPV61REF mapping	6996	SNV	C	A	496	25138	1.97	37.33	HPV61-L1:c.1233C>A	
140	LSIL	NC	HPV61REF mapping	7056	SNV	G	T	426	21158	2.01	37.20	HPV61-L1:c.1293G>T	HPV61-L1:p.Lys431Asn
140	LSIL	NC	HPV61REF mapping	7069	SNV	C	A	277	18194	1.52	37.12	HPV61-L1:c.1306C>A	HPV61-L1:p.Pro436Thr
140	LSIL	NC	HPV61REF mapping	7077	SNV	C	A	443	18723	2.37	37.02	HPV61-L1:c.1314C>A	
140	LSIL	NC	HPV61REF mapping	7154	SNV	T	A	2321	5299	43.80	36.22	HPV61-L1:c.1391T>A	HPV61-L1:p.Phe464Tyr
140	LSIL	NC	HPV61REF mapping	7158	SNV	T	C	76	455	16.70	34.89	HPV61-L1:c.1395T>C	
140	LSIL	NC	HPV61REF mapping	7158	MNV	TT	CC	7	456	1.54	35.14	HPV61-L1:c.1395_1396delTTinsCC	
140	LSIL	NC	HPV61REF mapping	7159	Insertion	-	C	2	176	1.14	35.50	HPV61-L1:c.1395_1396insC	HPV61-L1:p.Arg468fs
140	LSIL	NC	HPV61REF mapping	7159	SNV	T	C	22	176	12.50	33.82	HPV61-L1:c.1396T>C	
140	LSIL	NC	HPV62REF mapping	6739	SNV	T	C	40	157	25.48	35.93	HPV62-L1:c.963T>C	
140	LSIL	NC	HPV62REF mapping	6747	SNV	T	C	3	177	1.69	38.00	HPV62-L1:c.971T>C	HPV62-L1:p.Ile324Thr
140	LSIL	NC	HPV62REF mapping	6764	SNV	C	T	12	191	6.28	35.67	HPV62-L1:c.988C>T	
140	LSIL	NC	HPV62REF mapping	6790	SNV	T	C	5	235	2.13	31.80	HPV62-L1:c.1014T>C	
140	LSIL	NC	HPV62REF mapping	6793	SNV	G	A	4	231	1.73	31.25	HPV62-L1:c.1017G>A	
140	LSIL	NC	HPV62REF mapping	6864	SNV	T	A	3	207	1.45	38.00	HPV62-L1:c.1088T>A	HPV62-L1:p.Phe363Tyr
140	LSIL	NC	HPV62REF mapping	6871	SNV	A	C	3	205	1.46	38.00	HPV62-L1:c.1095A>C	
140	LSIL	NC	HPV62REF mapping	6874	SNV	C	T	3	211	1.42	38.00	HPV62-L1:c.1098C>T	
140	LSIL	NC	HPV62REF mapping	6877	SNV	G	A	8	214	3.74	36.88	HPV62-L1:c.1101G>A	
140	LSIL	NC	HPV62REF mapping	6883	SNV	A	G	6	196	3.06	37.50	HPV62-L1:c.1107A>G	
140	LSIL	NC	HPV62REF mapping	6905	SNV	C	A	5	212	2.36	36.60	HPV62-L1:c.1129C>A	HPV62-L1:p.Gln377Lys
140	LSIL	NC	HPV62REF mapping	6937	SNV	C	T	6	212	2.83	36.83	HPV62-L1:c.1161C>T	

140	LSIL	NC	HPV62REF mapping	6949	SNV	G	A	6	233	2.58	35.33	HPV62-L1:c.1173G>A	
140	LSIL	NC	HPV62REF mapping	6961	SNV	C	T	3	206	1.46	37.67	HPV62-L1:c.1185C>T	
140	LSIL	NC	HPV62REF mapping	6966	Deletion	A	-	4	192	2.08	33.75	HPV62-L1:c.1190delA	HPV62-L1:p.Asp397fs
140	LSIL	NC	HPV62REF mapping	6971	Insertion	-	G	8	197	4.06	37.38	HPV62-L1:c.1194_1195insG	HPV62-L1:p.Leu399fs
140	LSIL	NC	HPV62REF mapping	6994	MNV	TT	GG	2	195	1.03	38.00	HPV62-L1:c.1218_1219delTTinsGG	HPV62-L1:p.Leu407Val
140	LSIL	NC	HPV62REF mapping	7000	SNV	T	A	2	198	1.01	38.00	HPV62-L1:c.1224T>A	
140	LSIL	NC	HPV62REF mapping	7004	SNV	C	A	6	201	2.99	37.33	HPV62-L1:c.1228C>A	HPV62-L1:p.Pro410Thr
140	LSIL	NC	HPV62REF mapping	7022	SNV	G	A	4	212	1.89	37.00	HPV62-L1:c.1246G>A	HPV62-L1:p.Glu416Lys
140	LSIL	NC	HPV62REF mapping	7085	SNV	C	A	7	224	3.13	37.43	HPV62-L1:c.1309C>A	HPV62-L1:p.Pro437Thr
140	LSIL	NC	HPV62REF mapping	7090	SNV	G	T	6	223	2.69	37.33	HPV62-L1:c.1314G>T	HPV62-L1:p.Lys438Asn
140	LSIL	NC	HPV62REF mapping	7110	SNV	T	G	2	184	1.09	38.00	HPV62-L1:c.1334T>G	HPV62-L1:p.Met445Arg
140	LSIL	NC	HPV62REF mapping	7126	SNV	G	T	7	165	4.24	36.57	HPV62-L1:c.1350G>T	
140	LSIL	NC	HPV62REF mapping	7144	SNV	G	T	5	130	3.85	37.80	HPV62-L1:c.1368G>T	HPV62-L1:p.Leu456Phe
140	LSIL	NC	HPV62REF mapping	7147	SNV	T	C	6	121	4.96	36.67	HPV62-L1:c.1371T>C	
140	LSIL	CARC	HPV39REF mapping	6593	SNV	C	A	92	232	39.66	34.97	HPV39-L1:c.951C>A	
140	LSIL	CARC	HPV39REF mapping	6593	SNV	C	T	137	232	59.05	35.76	HPV39-L1:c.951C>T	
140	LSIL	CARC	HPV39REF mapping	6593	Deletion	C	-	3	232	1.29	38.00	HPV39-L1:c.952delC	HPV39-L1:p.His318fs
140	LSIL	CARC	HPV39REF mapping	6596	SNV	C	T	326	329	99.09	36.92	HPV39-L1:c.954C>T	
140	LSIL	CARC	HPV39REF mapping	6599	SNV	C	T	68	730	9.32	36.28	HPV39-L1:c.957C>T	
140	LSIL	CARC	HPV39REF mapping	6603	SNV	G	T	14	1025	1.37	36.86	HPV39-L1:c.961G>T	HPV39-L1:p.Gly321Cys
140	LSIL	CARC	HPV39REF mapping	6608	SNV	A	T	26	1228	2.12	36.69	HPV39-L1:c.966A>T	
140	LSIL	CARC	HPV39REF mapping	6651	SNV	C	A	40	1957	2.04	36.68	HPV39-L1:c.1009C>A	HPV39-L1:p.Arg337Ser
140	LSIL	CARC	HPV39REF mapping	6785	SNV	C	A	27	1774	1.52	36.93	HPV39-L1:c.1143C>A	
140	LSIL	CARC	HPV39REF mapping	6903	SNV	C	T	1602	1607	99.69	36.85	HPV39-L1:c.1261C>T	
140	LSIL	CARC	HPV39REF mapping	6939	SNV	C	A	23	1395	1.65	36.48	HPV39-L1:c.1297C>A	HPV39-L1:p.Pro433Thr
140	LSIL	CARC	HPV39REF mapping	6997	SNV	G	T	12	712	1.69	36.42	HPV39-L1:c.1355G>T	HPV39-L1:p.Arg452Met
140	LSIL	CARC	HPV39REF mapping	7025	SNV	A	G	309	323	95.67	37.09	HPV39-L1:c.1383A>G	
140	LSIL	CARC	HPV39REF mapping	7025	Replacement	A	GT	12	323	3.72	37.17	HPV39-L1:c.1383delAinsGT	HPV39-L1:p.Leu464fs
140	LSIL	CARC	HPV39REF mapping	7027	Insertion	-	A	125	322	38.82	36.14	HPV39-L1:c.1384_1385insA	HPV39-L1:p.Phe462fs
140	LSIL	CARC	HPV39REF mapping	7028	SNV	C	T	147	289	50.87	35.66	HPV39-L1:c.1386C>T	
141	LSIL	POSC	HPV53REF mapping	6601	SNV	A	T	191	218	87.61	37.30	HPV53-L1:c.944A>T	HPV53-L1:p.Gln315Leu
141	LSIL	POSC	HPV53REF mapping	6605	SNV	A	T	2284	8735	26.15	35.16	HPV53-L1:c.948A>T	
141	LSIL	POSC	HPV53REF mapping	6605	Deletion	A	-	175	8735	2.00	36.64	HPV53-L1:c.948delA	HPV53-L1:p.His317fs
141	LSIL	POSC	HPV53REF mapping	6611	SNV	T	C	4409	27802	15.86	36.35	HPV53-L1:c.954T>C	
141	LSIL	POSC	HPV53REF mapping	7033	SNV	T	A	5826	13326	43.72	36.35	HPV53-L1:c.1376T>A	HPV53-L1:p.Phe459Tyr
141	LSIL	POSC	HPV53REF mapping	7037	SNV	T	C	1491	2295	64.97	35.21	HPV53-L1:c.1380T>C	
141	LSIL	POSC	HPV53REF mapping	7037	Deletion	T	-	94	2295	4.10	34.44	HPV53-L1:c.1380delT	HPV53-L1:p.Gly462fs
141	LSIL	POSC	HPV53REF mapping	7040	SNV	T	G	15	189	7.94	34.33	HPV53-L1:c.1383T>G	
141	LSIL	NC	HPV61REF mapping	6720	SNV	C	A	90	197	45.69	35.34	HPV61-L1:c.957C>A	
141	LSIL	NC	HPV61REF mapping	6720	SNV	C	T	105	197	53.30	35.77	HPV61-L1:c.957C>T	
141	LSIL	NC	HPV61REF mapping	6720	Deletion	C	-	2	197	1.02	37.50	HPV61-L1:c.958delC	HPV61-L1:p.His320fs
141	LSIL	NC	HPV61REF mapping	6723	SNV	C	T	254	254	100.00	37.24	HPV61-L1:c.960C>T	
141	LSIL	NC	HPV61REF mapping	6726	SNV	C	T	16	441	3.63	37.19	HPV61-L1:c.963C>T	
141	LSIL	NC	HPV61REF mapping	6767	SNV	T	C	22	798	2.76	36.45	HPV61-L1:c.1004T>C	HPV61-L1:p.Val335Ala
141	LSIL	NC	HPV61REF mapping	6812	Insertion	-	C	32	1035	3.09	37.81	HPV61-L1:c.1055dup	HPV61-L1:p.Val353fs
141	LSIL	NC	HPV61REF mapping	6833	SNV	A	G	16	1050	1.52	37.25	HPV61-L1:c.1070A>G	HPV61-L1:p.Lys357Arg
141	LSIL	NC	HPV61REF mapping	6898	SNV	T	C	24	928	2.59	37.54	HPV61-L1:c.1135T>C	
141	LSIL	NC	HPV61REF mapping	6998	SNV	C	T	30	1204	2.49	37.07	HPV61-L1:c.1235C>T	HPV61-L1:p.Ser412Phe
141	LSIL	NC	HPV61REF mapping	7154	SNV	T	A	76	199	38.19	36.53	HPV61-L1:c.1391T>A	HPV61-L1:p.Phe464Tyr

141	LSIL	NC	HPV61REF mapping	7158	SNV	T	C	11	32	34.38	35.36	HPV61-L1:c.1395T>C	
160	LSIL	CARC	HPV16REF mapping	6585	Deletion	C	-	10	18	55.56	36.90	HPV16-L1:c.947delC	HPV16-L1:p.Ala316fs
160	LSIL	CARC	HPV16REF mapping	6588	SNV	A	G	19	71	26.76	36.89	HPV16-L1:c.950A>G	HPV16-L1:p.Gln317Arg
160	LSIL	CARC	HPV16REF mapping	6588	SNV	A	T	2	71	2.82	32.50	HPV16-L1:c.950A>T	HPV16-L1:p.Gln317Leu
160	LSIL	CARC	HPV16REF mapping	6589	Insertion	-	G	4	71	5.63	38.00	HPV16-L1:c.953dup	HPV16-L1:p.His319fs
160	LSIL	CARC	HPV16REF mapping	6592	SNV	C	A	7736	18672	41.43	35.07	HPV16-L1:c.954C>A	
160	LSIL	CARC	HPV16REF mapping	6592	SNV	C	T	10804	18672	57.86	35.15	HPV16-L1:c.954C>T	
160	LSIL	CARC	HPV16REF mapping	6595	SNV	C	T	28483	28547	99.78	36.66	HPV16-L1:c.957C>T	
160	LSIL	CARC	HPV16REF mapping	6598	SNV	T	C	8236	46849	17.58	36.31	HPV16-L1:c.960T>C	
160	LSIL	CARC	HPV16REF mapping	6613	SNV	G	T	824	59825	1.38	37.12	HPV16-L1:c.975G>T	HPV16-L1:p.Trp325Cys
160	LSIL	CARC	HPV16REF mapping	6959	SNV	C	A	828	66119	1.25	37.21	HPV16-L1:c.1321C>A	HPV16-L1:p.Leu441Ile
160	LSIL	CARC	HPV16REF mapping	7020	SNV	A	C	336	28733	1.17	36.86	HPV16-L1:c.1382A>C	HPV16-L1:p.Gln461Pro
160	LSIL	CARC	HPV16REF mapping	7023	SNV	T	A	10456	25749	40.61	35.89	HPV16-L1:c.1385T>A	HPV16-L1:p.Phe462Tyr
160	LSIL	CARC	HPV16REF mapping	7027	SNV	T	C	197	1108	17.78	35.45	HPV16-L1:c.1389T>C	
160	LSIL	CARC	HPV16REF mapping	7027	MNV	TT	CC	15	1109	1.35	36.50	HPV16-L1:c.1389_1390delTTinsCC	
160	LSIL	CARC	HPV16REF mapping	7028	SNV	T	C	6	309	1.94	37.00	HPV16-L1:c.1390T>C	
160	LSIL	CARC	HPV16REF mapping	7030	SNV	A	G	8	28	28.57	37.50	HPV16-L1:c.1392A>G	
160	LSIL	CARC	HPV16REF mapping	7030	SNV	A	T	19	28	67.86	36.74	HPV16-L1:c.1392A>T	HPV16-L1:p.Leu464Phe
160	LSIL	CARC	HPV18REF mapping	6566	SNV	T	A	94	553	17.00	35.60	HPV18-L1:c.954T>A	
160	LSIL	CARC	HPV18REF mapping	6572	SNV	C	T	87	1423	6.11	36.03	HPV18-L1:c.960C>T	
160	LSIL	CARC	HPV18REF mapping	6834	Insertion	-	C	38	2321	1.64	35.61	HPV18-L1:c.1229dup	HPV18-L1:p.Pro411fs
160	LSIL	CARC	HPV18REF mapping	6834	Deletion	C	-	48	2318	2.07	34.98	HPV18-L1:c.1229delC	HPV18-L1:p.Pro410fs
160	LSIL	CARC	HPV18REF mapping	6839	SNV	C	A	26	2288	1.14	36.54	HPV18-L1:c.1227C>A	
160	LSIL	CARC	HPV18REF mapping	6841	SNV	C	A	26	2257	1.15	35.96	HPV18-L1:c.1229C>A	HPV18-L1:p.Pro410Gln
160	LSIL	CARC	HPV18REF mapping	6935	SNV	C	A	25	1799	1.39	37.72	HPV18-L1:c.1323C>A	
160	LSIL	CARC	HPV18REF mapping	6984	MNV	TT	GC	28	1028	2.72	36.54	HPV18-L1:c.1372_1373delTTinsGC	HPV18-L1:p.Leu458Ala
160	LSIL	CARC	HPV18REF mapping	6990	SNV	T	C	26	887	2.93	37.12	HPV18-L1:c.1378T>C	
160	LSIL	CARC	HPV18REF mapping	6998	SNV	A	G	545	545	100.00	36.54	HPV18-L1:c.1386A>G	
160	LSIL	CARC	HPV18REF mapping	7000	SNV	A	T	111	546	20.33	35.45	HPV18-L1:c.1388A>T	HPV18-L1:p.Tyr463Phe
160	LSIL	CARC	HPV18REF mapping	7004	SNV	C	T	5	385	1.30	34.80	HPV18-L1:c.1392C>T	

Avg, average; Carc, carcinogenicity; Freq, frequency; IARC, International Agency for Research on Cancer; ID, identification; Pap, Papanicolau smear; Ref, reference; Ref A, reference allele

[a]Variant Table Definititions (CLC Genomics Workbench 11.0.1)

Variant type: The type of variant. This can either be SNV (single-nucleotide variant), MNV (multi-nucleotide variant), insertion, deletion, or replacement.

Reference: The reference sequence at the position of the variant.

Allele: The allele sequence of the variant.

Reference allele: Describes whether the variant is identical to the reference.

Count: The number of 'countable' reads supporting the allele.

Coverage: The read coverage at this position. Only 'countable' reads are considered.

Frequency: The number of 'countable' reads supporting the allele divided by the number of 'countable' reads covering the position of the variant.

Mapping: Reference sequence or genome used for mapping.
