# Supplementary material for: Abundance of HPV L1 Intra-Genotype Variants With Capsid Epitopic Modifications Found Within Low- and High-Grade Pap Smears With Potential Implications for Vaccinology
Source: Front Genet. 2019 May 24;10:489. doi: 10.3389/fgene.2019.00489 (PMC6558378; doi:10.3389/fgene.2019.00489)
Supplement: Supplementary file 2 [file Data_Sheet_2.PDF]

A

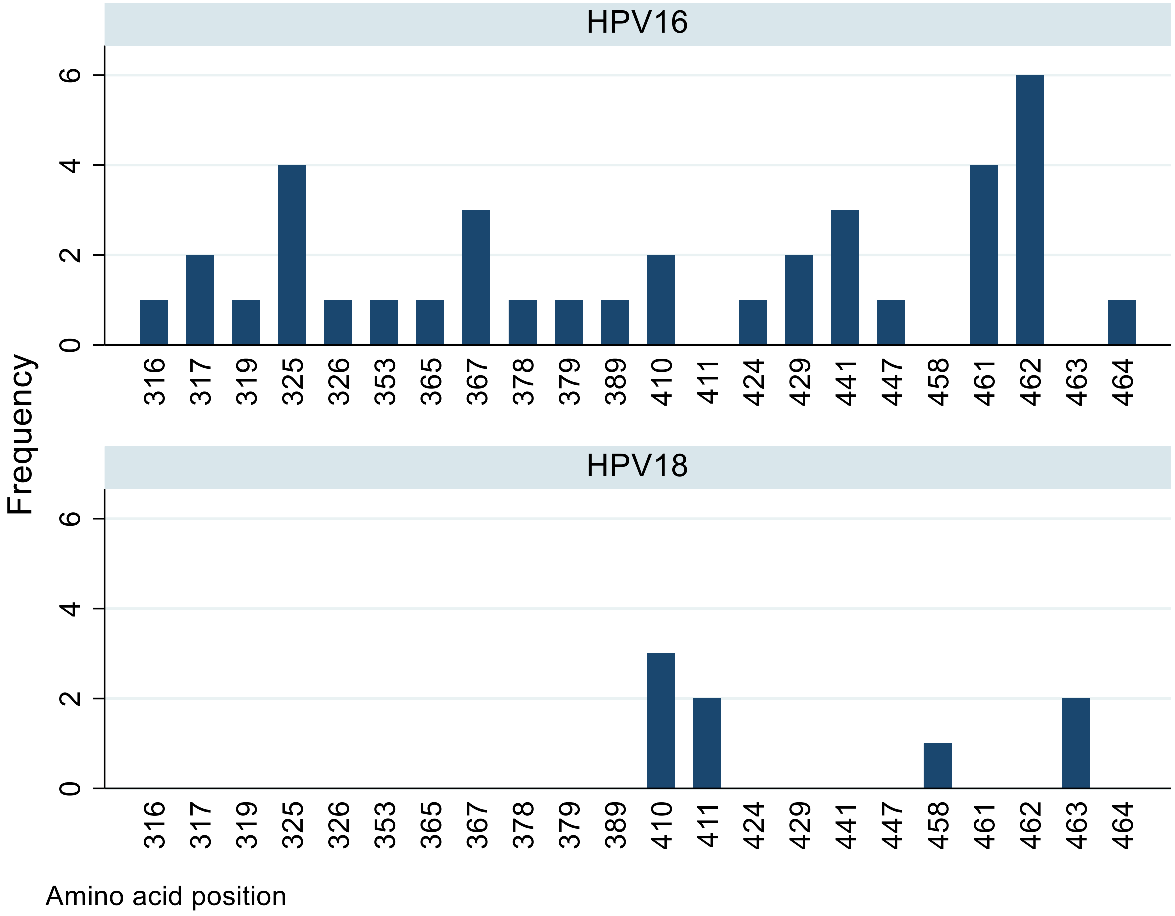
HPV16
HPV18
Frequency
0
2
4
6
316 317 319 325 326 353 365 367 378 379 389 410 411 424 429 441 447 458 461 462 463 464
Amino acid position

B

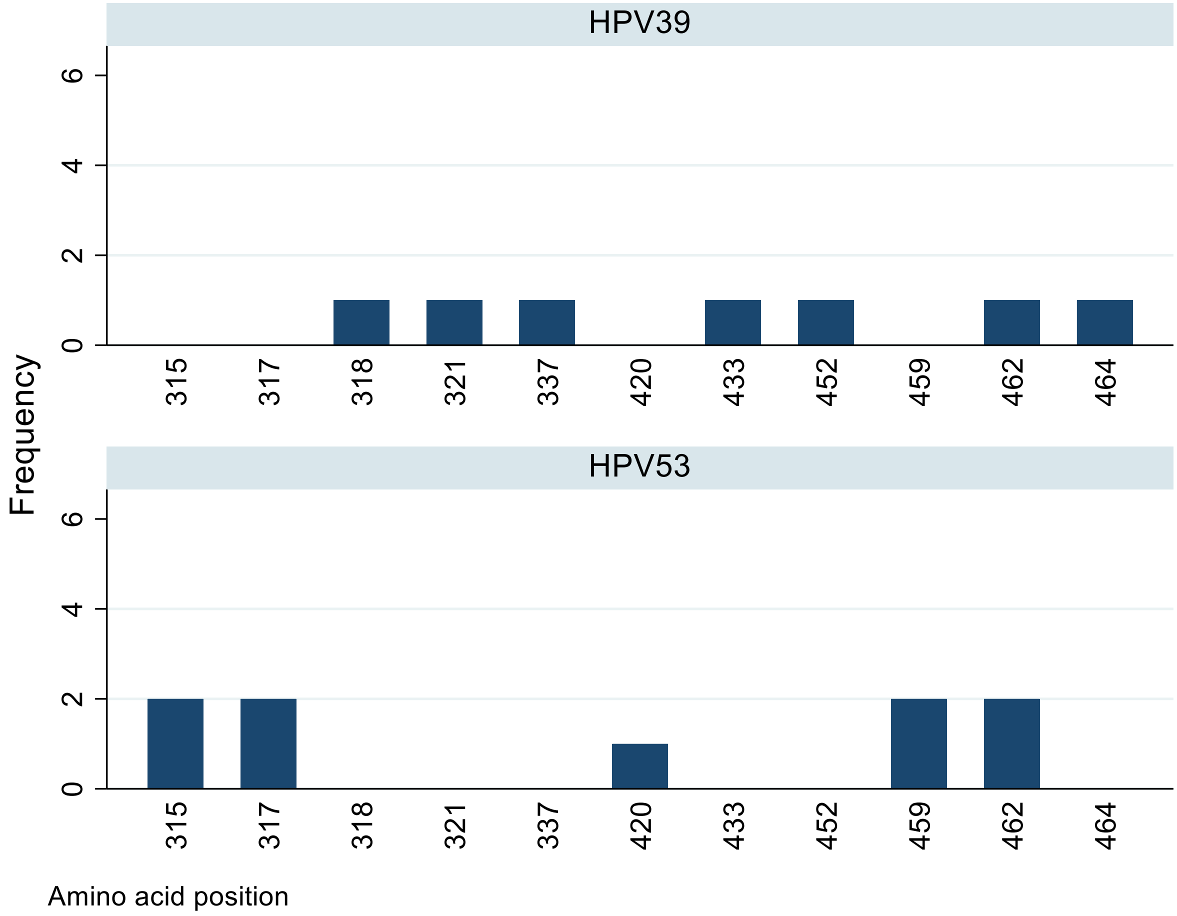
HPV39
HPV53
Frequency
0
2
4
6
315
317
318
321
337
420
433
452
459
462
464
Amino acid position

C

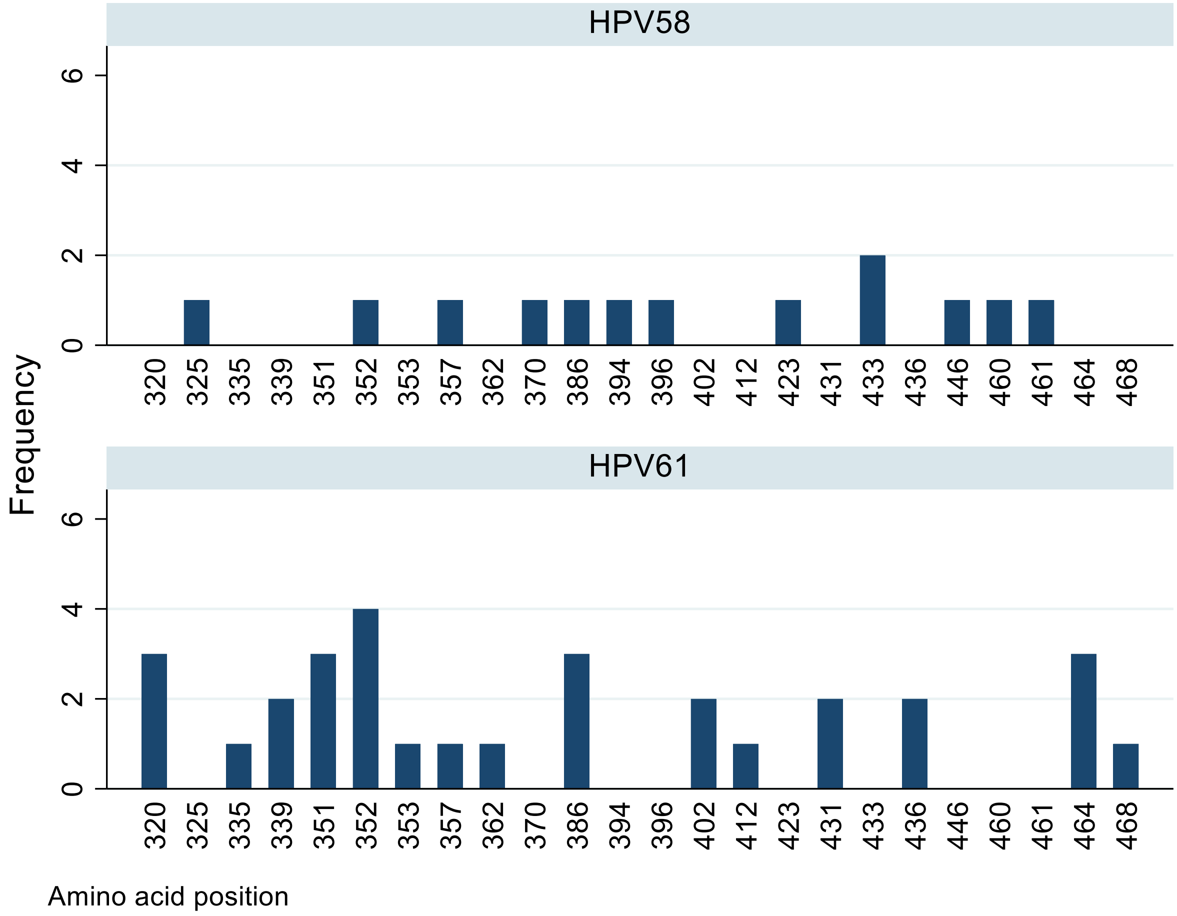
HPV58
HPV61
Frequency
0
2
4
6
320
325
335
339
351
352
353
357
362
370
386
394
396
402
412
423
431
433
436
446
460
461
464
468
Amino acid position

D

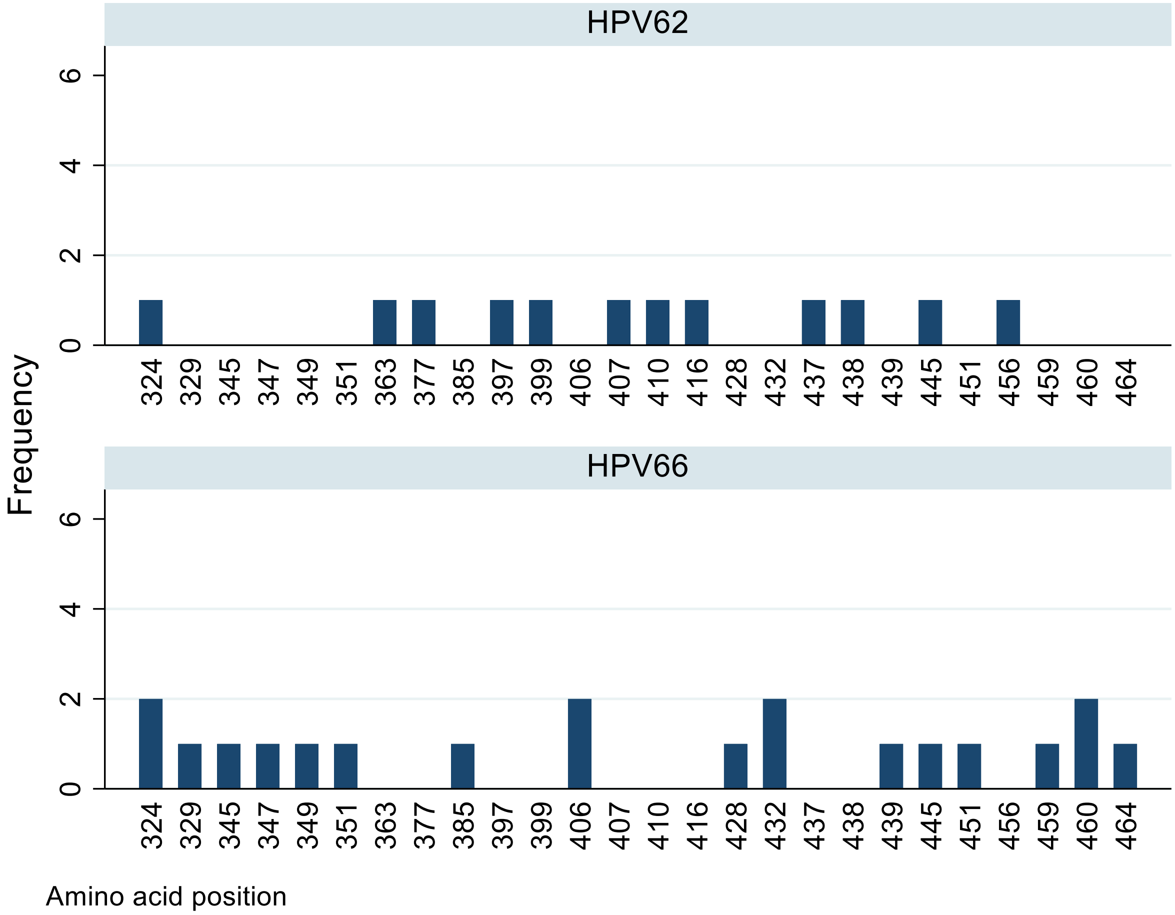
HPV62
HPV66
Frequency
0
2
4
6
324
329
345
347
349
351
363
377
385
397
399
406
407
410
416
428
432
437
438
439
445
451
456
459
460
464
Amino acid position

E

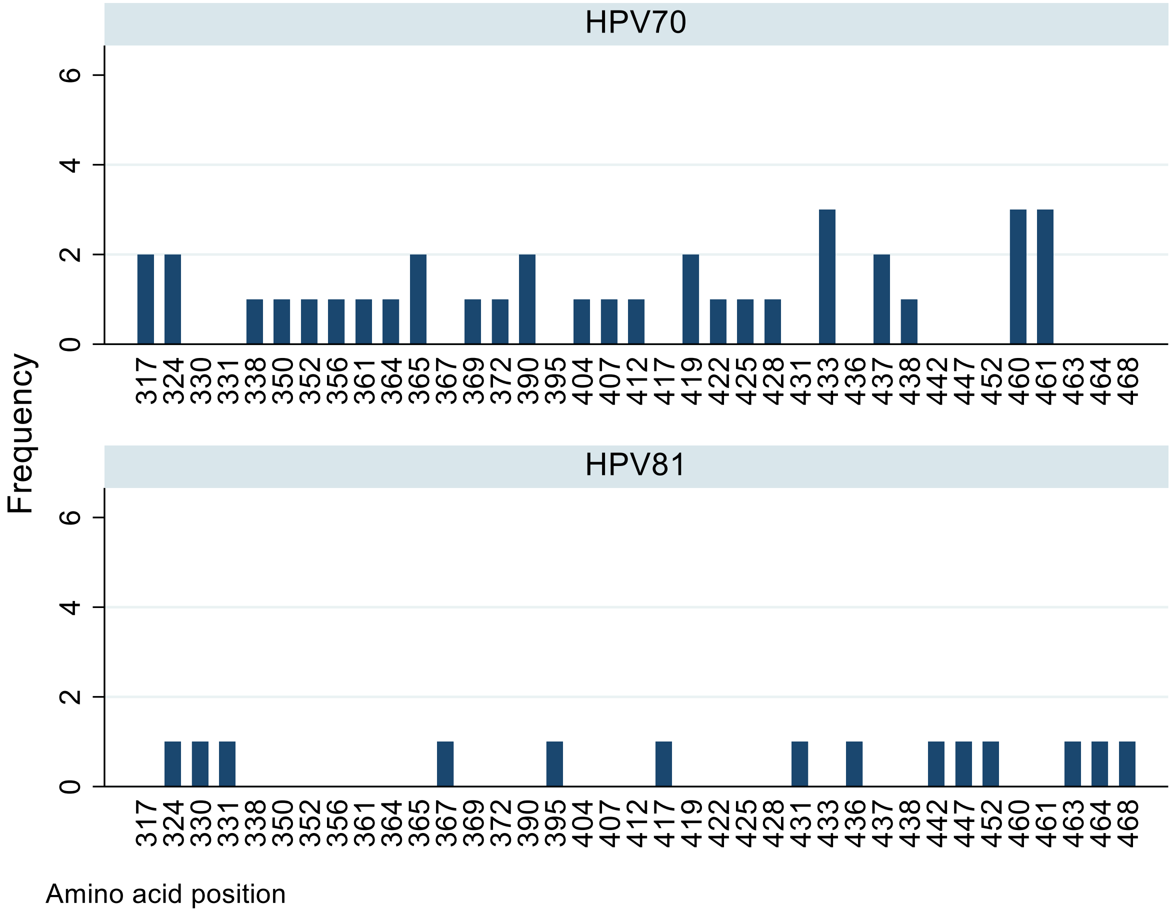
HPV70
HPV81
Frequency
0
2
4
6
317 324 330 331 338 350 352 356 361 364 365 367 369 372 390 395 404 407 412 417 419 422 425 428 431 433 436 437 438 442 447 452 460 461 463 464 468
Amino acid position

F

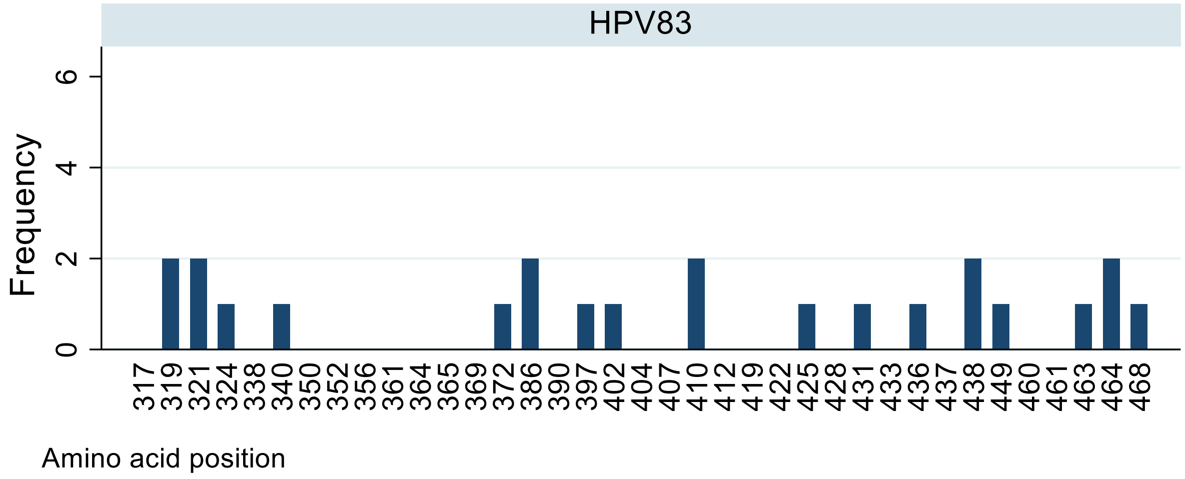

Supplementary Figure 1. Distribution of variants in HPV L1 by amino acid positions according to HPV genotype. Patterns of variants for: (**A**) HPV-16, 18, (**B**) 39, 53, (**C**) 58, 61, (**D**) 62, 66, (**E**) 70, 81, and (**F**) 83 are different and may be mapped to L1 hypervariable loops for identification of epitopic modifications of significance.
