# Supplementary material for: Abundance of HPV L1 Intra-Genotype Variants With Capsid Epitopic Modifications Found Within Low- and High-Grade Pap Smears With Potential Implications for Vaccinology
Source: Front Genet. 2019 May 24;10:489. doi: 10.3389/fgene.2019.00489 (PMC6558378; doi:10.3389/fgene.2019.00489)
Supplement: Supplementary file 3 [file Data_Sheet_3.PDF]

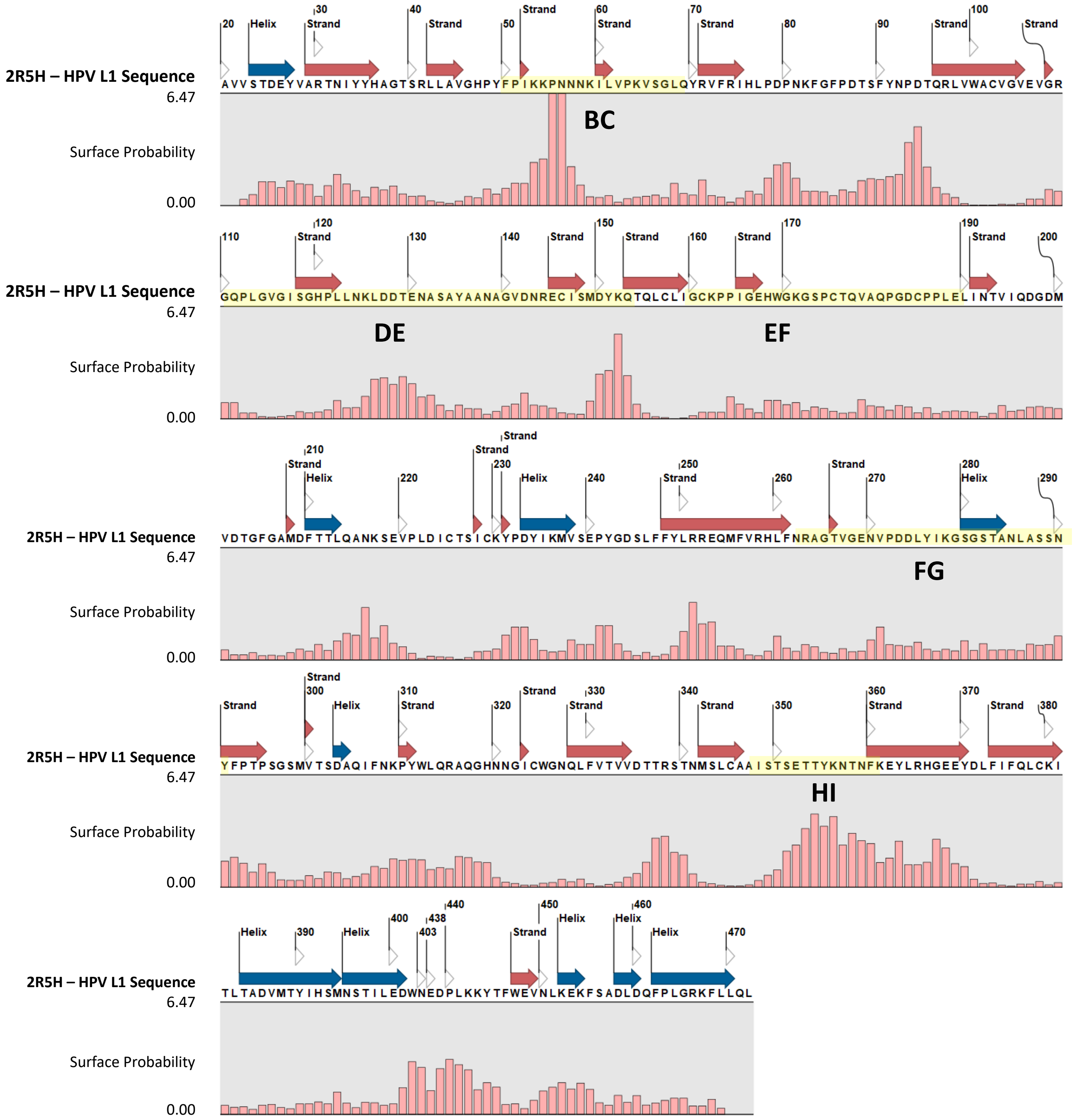

Supplementary Figure 2. HPV-16 L1 major capsid protein sequence with surface probability plot reconstructed from annotated sequences available in Protein Data Bank (PDB ID: 2R5H) (Bishop et al. 2007). The regions with higher surface probabilities (pink bars) correlates well with the known antigenic hypervariable loops: BC (AA 50-69), DE (AA 110-153), EF (AA 160-189), FG (AA 262-291), and HI (AA 348-360) (amino acid sequence highlighted in yellow). The surface probability is based on the algorithm by Emini et al., 1985 for prediction of antigenic determinants on surface of proteins with an estimated accuracy of 75%.
