# Supplementary material for: Abundance of HPV L1 Intra-Genotype Variants With Capsid Epitopic Modifications Found Within Low- and High-Grade Pap Smears With Potential Implications for Vaccinology
Source: Front Genet. 2019 May 24;10:489. doi: 10.3389/fgene.2019.00489 (PMC6558378; doi:10.3389/fgene.2019.00489)
Supplement: Supplementary file 4 [file Data_Sheet_4.PDF]

A

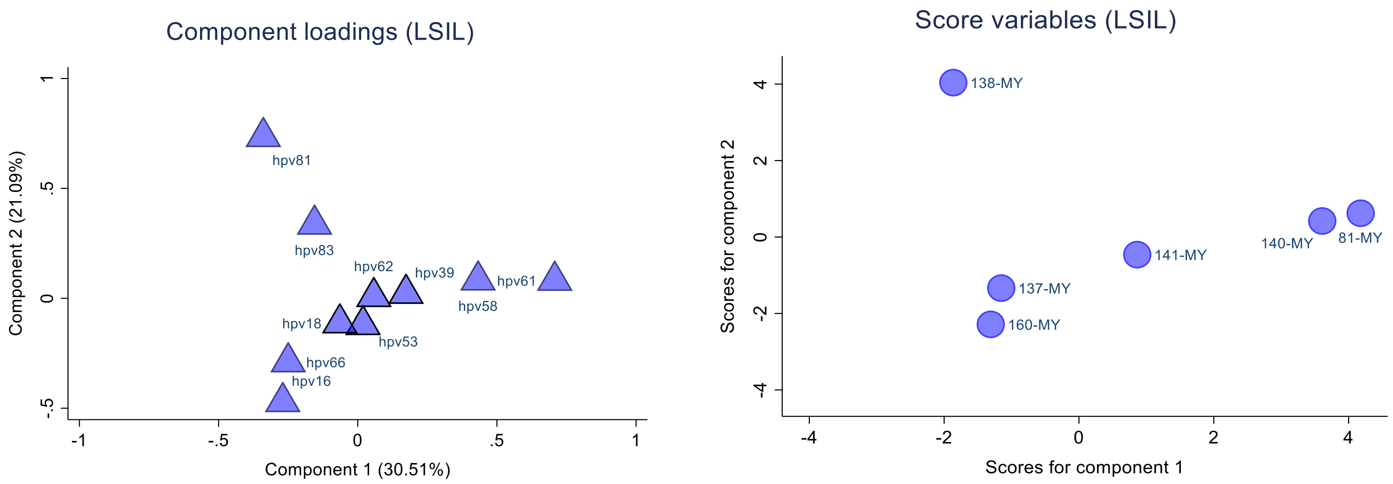

B

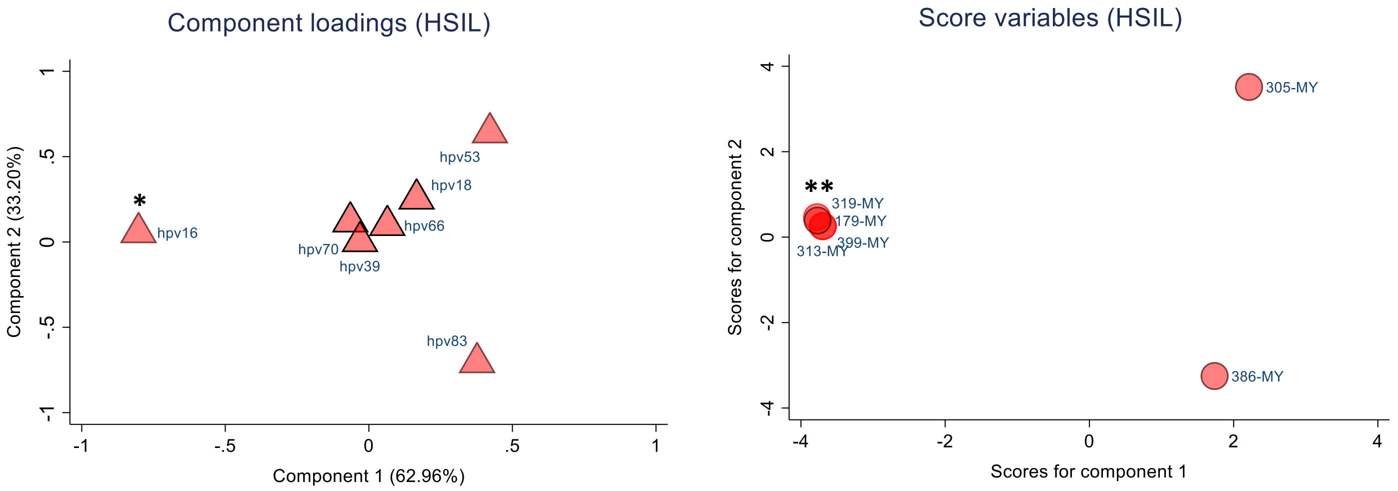

Supplementary Figure 3. Principal component analysis (PCA) of HPV genotype composition in LSIL (A) and HSIL (B) samples. The Component loadings and Score variables plots show the components from the perspective of the variables or the observations, respectively. The component loadings plot for LSIL and HSIL show the correlative relationship between HPV genotypes along the first two principal components axes (PC1 and PC2). The sum of PC1 and PC2 explain 51.6% and 96.2% of the total variance for LSIL and HSIL, respectively. Comparing LSIL and HSIL, HPV-16 (*) emerged from all other genotypes as the dominant component in HSIL. The score variables plots display each sample's contribution to the principal components. HSIL compared to LSIL had a preponderance of samples (**) containing a high composition of HPV-16. PCA was performed on the covariance matrix of natural log-transformed abundance data [ln (n +1)] of HPV genotypes within each sample. LSIL, low-grade squamous intraepithelial lesion; HSIL, high-grade squamous intraepithelial lesion.
